# The Malacca Strait separates distinct faunas of poorly-flying *Cautires* net-winged beetles

**DOI:** 10.7717/peerj.6511

**Published:** 2019-03-05

**Authors:** Alice Jiruskova, Michal Motyka, Matej Bocek, Ladislav Bocak

**Affiliations:** Laboratory of Molecular Systematics, Department of Zoology, Faculty of Science, Palacky University, Olomouc, Czech Republic

**Keywords:** Oriental region, Molecular phylogeny, Colonization, Mimicry

## Abstract

We investigated the spatial and temporal patterns of *Cautires* diversification on the Malay Peninsula and Sumatra to understand if the narrow and frequently dry Malacca Strait separates different faunas. Moreover, we analyzed the origin of *Cautires* in Malayan and Sumatran mountains. We sampled 18 localities and present the mtDNA-based phylogeny of 76 species represented by 388 individuals. The phylogenetic tree was dated using mtDNA evolution rates and the ancestral ranges were estimated using the maximum likelihood approach. The phylogeny identified multiple lineages on the Malay Peninsula since the Upper Eocene (35 million years ago, mya) and a delayed evolution of diversity in Sumatra since the Upper Oligocene (26 mya). A limited number of colonization events across the Malacca Strait was identified up to the Pliocene and more intensive faunal exchange since the Pleistocene. The early colonization events were commonly followed by in situ diversification. As a result, the Malacca Strait now separates two faunas with a high species-level turnover. The montane fauna diversified in a limited space and seldom took part in colonization events across the Strait. Besides isolation by open sea or a savannah corridor, mimetic patterns could decrease the colonization capacity of *Cautires*. The Malay fauna is phylogenetically more diverse and has a higher value if conservation priorities should be defined.

## Introduction

Geographic isolation is an important factor in the speciation process (Lester et al., 2007; Barraclough & Vogler, 2000) and recent studies have shown that poor dispersers have a tendency to produce a higher number of species in a small area (Ikeda, Nishikawa & Sota, 2012; Bray & Bocak, 2016). The number of individuals which are able to cross a geographical barrier depends on the dispersal propensity of the animals under consideration; for example, flightless species have a much lower chance to cross a sea barrier than highly mobile long-distance flying insects (Emerson, Oromi & Hewitt, 2000; Yoder & Nowak, 2006; Lohman et al., 2011; Husemann, Deppermann & Hochkirch, 2014; Toussaint et al., 2015, 2017b; Bray & Bocak, 2016; Bocek et al., 2018). Most beetles (Coleoptera) are winged and many of them are able to fly over long distances, especially those depending on ephemeral habitats or food sources, like lentic water beetles and coprophagous beetles. Their ability to frequently cross wide sea straits and to establish permanent populations was demonstrated in numerous phylogeographic studies (Balke et al., 2009; Toussaint et al., 2015; Tseng et al., 2018). Further studies addressed colonization and diversification on oceanic (Bell et al., 2015; Husemann, Deppermann & Hochkirch, 2014) and continental islands (Michaelides et al., 2015; Fuchs et al., 2016). Based on these studies, we can expect low turnover in the flying insects of geographically close and repeatedly connected landmasses.

**Figure 1:**
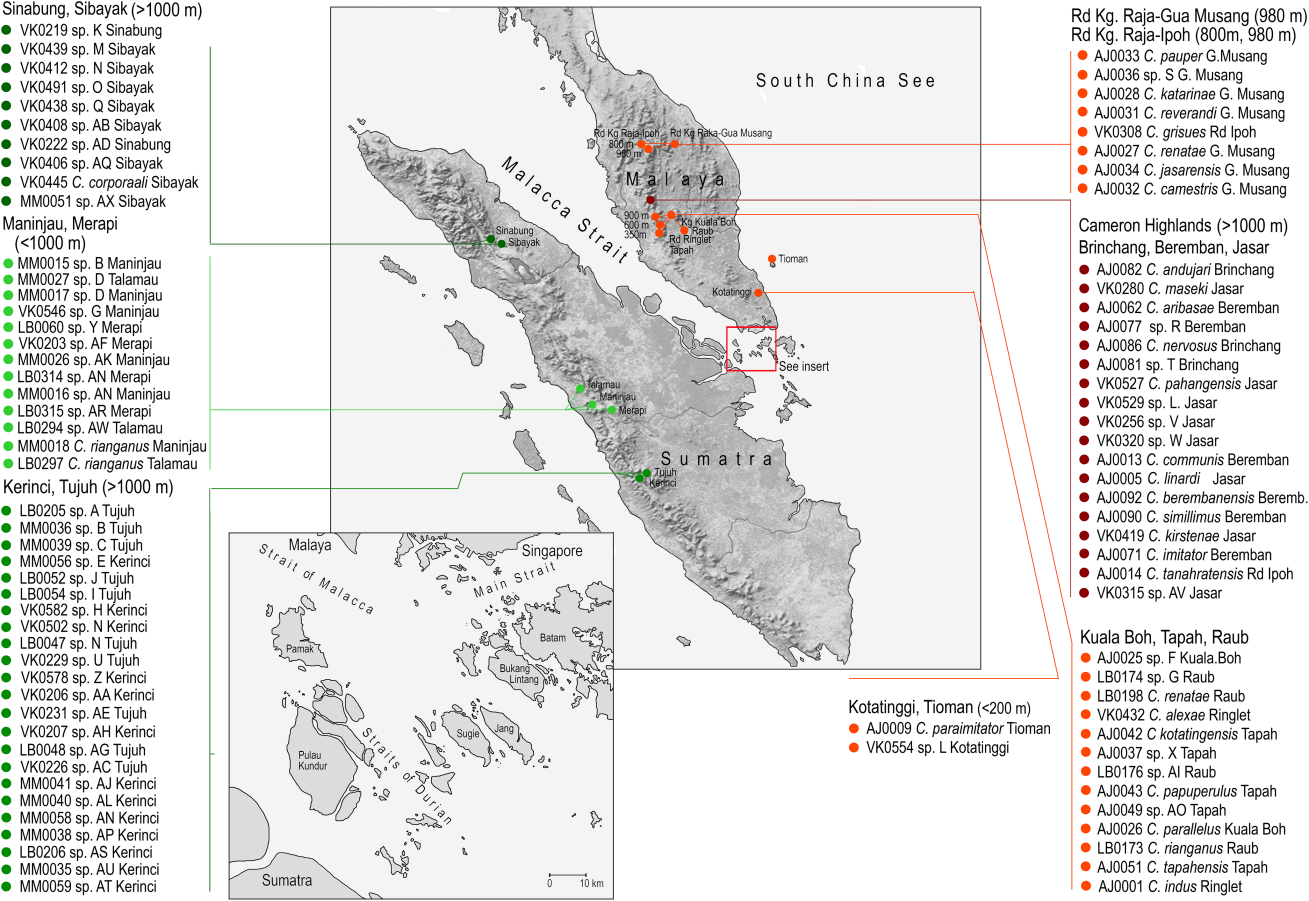
Study area. The sampled localities with the list of species collected in each locality.

Our study area is located in the western part of the Sunda Shelf which includes the Malay Peninsula and Sumatra separated by the shallow and narrow Malacca Strait (Hall, 2002; Cottam, Hall & Ghani, 2013). Its present width is 40–150 km and the depth up to 120 m. The southernmost part is packed with small islands separated by a maximum distance eight km at the present sea level and with an extensive dry-land if the sea level is only a few meters lower (Fig. 1; http://maps.ngdc.noaa.gov/viewers/bathymetry/; Voris, 2000). Compared to widely accepted zoogeographical boundaries (e.g., Wallace’s, Weber’s and Lydekker’s lines), the Strait has never been considered as a serious barrier for a faunal exchange (Mayr, 1944; Lohman et al., 2011). The Malay Peninsula and Sumatra have a different tectonic history. Unlike the tectonically very stable Malay Peninsula with old, eroded land blocks, Sumatra is a geologically dynamic region and was partly submerged and disintegrated into a number of smaller islands in the Upper Oligocene and Lower Miocene. These isolated islands became a single landmass about 15 million years ago (mya; Hall, 2002). There is no information about the extent of dry land left when Sumatra was submerged (Hall, 2002), but the presence of ancient neotenic lineages indicates that several parts were not inundated (Malohlava & Bocak, 2010; Masek et al., 2014).

Now, the predominant ecosystems in the Sundaland are humid rainforests and it is supposed that they have been present in the area since the origin of the Asian monsoon circulation associated with the uplift of the Himalayas ∼50 mya (Heaney, 1991). Knowledge of the earlier distribution of rainforests is limited, but the Pleistocene cold periods are known for their dry climate. During the glacial maxima, tropical forests shrank and mostly persisted in the mountains along the western coast of Sumatra and on the Malay Peninsula. Internal lowlands were covered by savannahs which are supposed to be a significant barrier to rainforest species (Heaney, 1991; Gathorne-Hardy et al., 2002; Lohman et al., 2011).

The Malay Peninsula and Sumatra represent a single zoogeographic region with a high number of widespread species (Myers et al., 2000). Nevertheless, some species are restricted to a limited part of the Sunda Shelf and their evolutionary history and distribution can elucidate faunal exchange and speciation history in South East Asia, as was demonstrated in recent studies of the great apes (Nater et al., 2017) or shrews (Demos et al., 2016). We examine phylogenetic relationships within the net-winged beetle genus *Cautires* Waterhouse, 1879 (Lycidae: Metriorrhynchini: Cautirina). These beetles are flight capable, but due to weak sclerotization, they fly slowly and usually only under the forest canopy (Linsley, Eisner & Klots, 1961; field observation). As they do not take food in the adult stage, they live for a short time, typically a few weeks. Additionally, their soft, highly permeable integument makes them sensitive to salt water and wide sea straits, such as the Makassar Strait, separate different faunas even at tribe levels despite the presence of large rivers which can bring a high amount of drifting debris and insects in the sea during torrential rains and flooding (Bocak, Matsuda & Yagi, 2006; Sklenarova, Chesters & Bocak, 2013; Masek et al., 2018). Although their colonization capacity has not been studied, the earlier published phylogenies have shown that net-winged beetle faunas with high species turnover can be separated by narrow sea straits (Malohlava & Bocak, 2010; Li, Bocak & Pang, 2015a; Li et al., 2015b; Li, Pang & Bocak, 2017b; Masek et al., 2018) and only some flower-visiting net-winged beetles are more effective colonists (Motyka, Masek & Bocak, 2017). Due to biological characteristics, *Cautires* have a lower dispersal capacity than long living, well-sclerotized and well-flying beetles such as water or dung beetles (Balke et al., 2009; Masek et al., 2014, 2015; Toussaint et al., 2015; Li, Pang & Bocak, 2017a ; Bocek et al., 2018). The genus was supposedly brought to Asia with drifting India 35–55 mya, its diversification started before the colonization of the Sunda Shelf and resulted in ∼170 described species from the Oriental region (Kleine, 1933; Bocak, 2002; Dudkova & Bocak, 2010; Sklenarova, Chesters & Bocak, 2013; Sklenarova, Kubecek & Bocak, 2014). The 53 Malay and Sumatran species represent a subset of Oriental *Cautires*. Morphological taxonomic studies have already identified high diversity, especially in the Malay montane forests where *Cautires* are more abundant compared to seasonally dry lowland ecosystems. Most species have restricted ranges and they do not occur across a wide range of elevations (Jiruskova & Bocak, 2015; Jiruskova, Motyka & Bocak, 2016). *Cautires*, as all net-winged beetles, are protected by smelly and bitter compounds in their hemolymph and they are usually aposematically colored and commonly mimicked by palatable insects (Linsley, Eisner & Klots, 1961; Eisner, Kafatos & Linsley, 1962; Eisner et al., 2008; Moore & Brown, 1981; Guilford et al., 1987; Lingafelter, 2013). Most aposematic patterns occur in clearly defined ranges and potentially prevent easy colonization of areas with different aposematic signals (Chouteau & Angers, 2011; Bocak & Yagi, 2010; Motyka, Kampova & Bocak, 2018).

The aim of this study is to investigate (1) the diversification of *Cautires* in the Sunda Shelf, (2) whether the narrow and repeatedly dry Malacca Strait separates different faunas and (3) whether turnover between neighboring Sumatra and the Malay Peninsula is produced by in situ speciation. If the Malacca Strait limits the number of successful colonization events, we should observe the clades that continually diversified either within Sumatra or Malaya. Alternatively, if *Cautires* frequently crossed the Malacca Strait or savannah ecosystems which replaced the strait during the Quaternary low-stand periods, we should identify widespread species with high intraspecific genetic variability. Additionally, multiple colonization events should be inferred within the clades of closely related species.

## Methods

### Sampling and sequencing

*Cautires* net-winged beetles from the Malay Peninsula and Sumatra were included in the dataset (Table S1). The available material contained 140 samples from Sumatra and 248 samples from the Malay Peninsula. The samples were collected in 18 localities from lowlands to 2,400 m above sea level (Fig. 1; Table S1). The collecting was approved by the permit No TS/PTD/5/4Jld48(41); some material was collected on public land, outside protected areas, no protected species were collected.

The total DNA was extracted from metathoracic muscles using the DNeasy tissue kit (Qiagen N.V., Venlo, Netherlands). Due to financial constraints that limit the genetic sequencing of hundreds of samples and the problem of identifying suitable genomic markers for the species-level phylogeny, only mitochondrial fragments were amplified: *rrnL*–tRNA-Leu–*nad1* (∼810 bp), the 3′end of *cox1*–tRNA-Leu–*cox2* (∼1,100 bp), and *nad5*–tRNA-Phe–tRNA-Glu–tRNA-Ser (∼1,310 bp). The primers and PCR conditions followed Sklenarova, Chesters & Bocak (2013). The PCR products were purified using PCRμ96 Plates (EMD Millipore Co., Burlington, MA, USA) and sequenced by an ABI 3130 automated sequencer using the Big Dye Sequencing Kit 1.1 (Thermo Fisher Scientific Inc., Foster City, CA, USA). The chromatograms produced by Sanger sequencing were edited using Sequencher 4.8 (Gene Codes Inc., Ann Arbor, MI, USA) and the new data (GenBank accession codes AB123456–AB123456, Table S1) were aligned with the previously published sequences representing several Metriorrhynchini genera as an outgroup (Table S2; Sklenarova, Chesters & Bocak, 2013; Sklenarova, Kubecek & Bocak, 2014).

### Species delimitation, phylogenetic analyses, reconstruction of ancestral areas and dating

The taxonomy of South East Asian *Cautires* has not been revised and original descriptions are uninformative. Only the Malay fauna was recently studied (Jiruskova & Bocak, 2015; Jiruskova, Motyka & Bocak, 2016). Therefore, we could not formally identify many Sumatran species and we had to delimit them here. We did not have a chance to test intrinsic reproductive isolation. Therefore, using the biological species concept (*sensu* Mayr 1942, see De Queiroz, 2005 for further discussion), we hypothesized that the sets of individuals which differ morphologically from other individuals are intrinsically reproductively isolated and represent separate biological species. We used external morphology, that is, the relative size of eyes in males, coloration, the shape of the pronotum, elytral cells and elytral costae. The characters were studied using a binocular microscope Olympus SXZ-16 under magnification 6–100×. Further, the genitalia of all species were dissected and cleaned from muscles and fat bodies to observe detailed structures. Genitalia often serve as a reproductive isolating mechanism and closely related net-winged beetle species regularly differ in their morphology (Malohlava & Bocak, 2010; Bocak & Yagi, 2010; Masek et al., 2015; Fig. S1). Further, we used mitochondrial *rrnL*, *cox1* and *nad5* markers to identify genetic differentiation between morphology-based species (Table S3). Although maternally inherited, these markers are commonly used to identify species limits (Ahrens et al., 2016, but Baselga et al., 2013). The comparison of color patterns, morphology and molecular differentiation can identify whether some species are color polymorphic. If we identified a set of individuals with highly similar morphology and mtDNA sequence, the difference in coloration was not considered as a proof of intrinsic reproductive isolation and we designated the individuals belonging to various color pattern subsets as a single putative biological species.

All individuals were dry-mounted and the color pattern of each individual was described and each individual was assigned to one of the following groups: (1) the pronotum and elytra completely black; (2) the pronotum and the humeral part of elytra light brown; (3) the pronotum red, humeri or at least the humeral part of elytral costae red; (4) the pronotum black, the basal part of elytra brightly colored, that is, red or brown; (5) the pronotum brightly orange, at least the humeral part of elytral costae brightly orange or whole elytra orange; (6) the pronotum black, the humeral part of elytra black, their apical part red; (7) the whole upper side of the body yellow; (8) the pronotum completely red or with a black patch in the middle, elytra black. The geographic distribution of color patterns was mapped.

Mitochondrial DNA fragments were separately aligned with MAFFT 7.017 plug-in (Katoh & Standley, 2013) in Geneious R7.1.9 (Biomatters Inc., Newark, NJ, USA) and G-Ins-i algorithm. The alignments of the protein-coding genes *cox1*, *cox2*, *nad1* and *nad5* were checked by amino acid reading frames and manually corrected where necessary. The *rrnL* fragment has a complex loop structure and its alignment may be more complicated if loops are extensive (Zhong & Zhang, 2012). The longest identified indel contained four positions, and therefore, we did not use a structural alignment approach to assess the homology of individual nucleotides and gaps. The concatenated supermatrix was analyzed under the maximum likelihood (ML) criterion using IQ-TREE 1.6.0 (Nguyen et al., 2015) with 5,000 UFboot iterations and partitioned by genes. Optimal models of evolution were identified by ModelFinder (Kalyaanamoorthy et al., 2017) implemented in IQ-TREE (Table S2).

**Figure 2:**
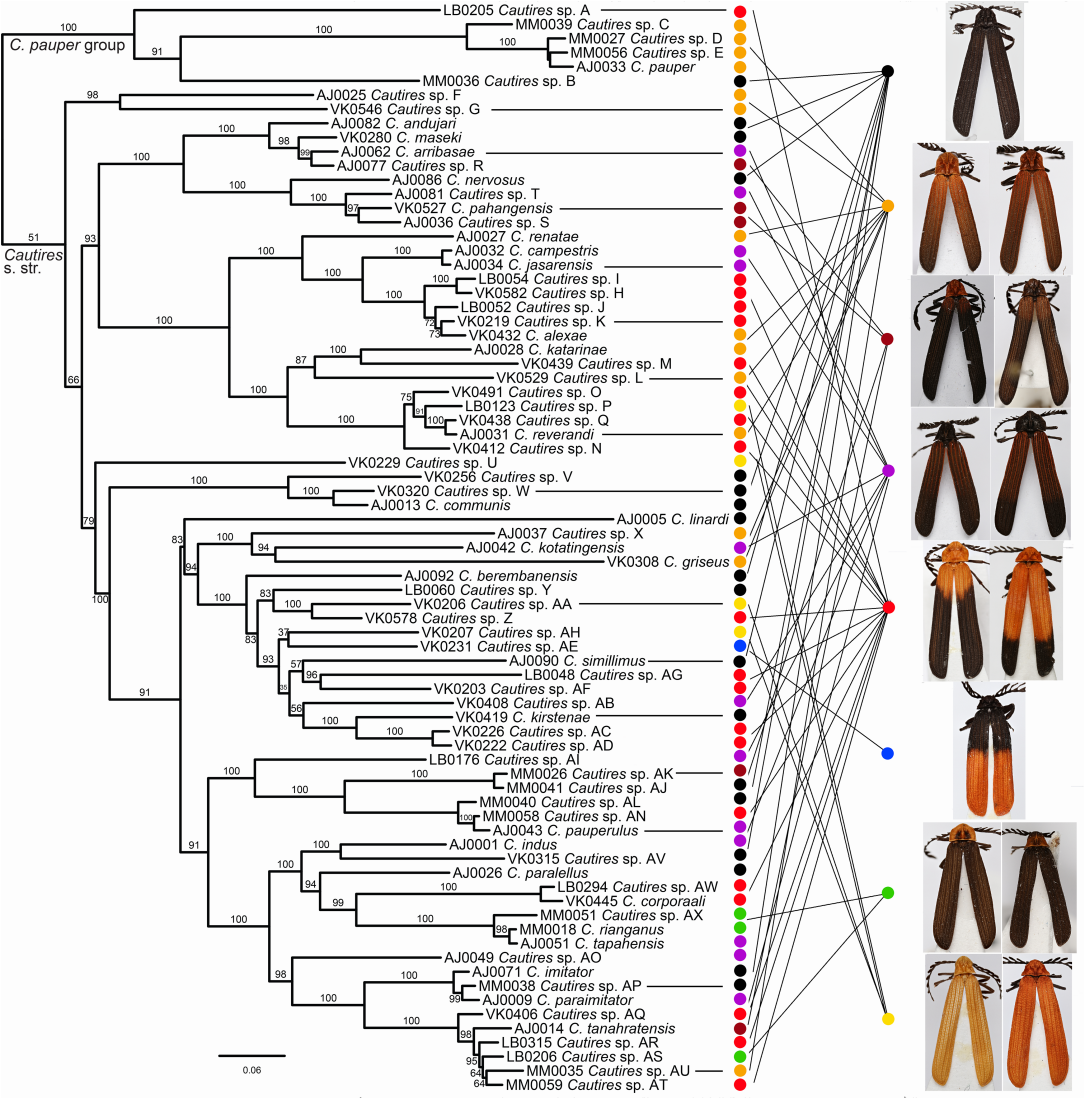
Phylogeny of *Cautires*. The maximum likelihood tree for *Cautires* recovered from the complete dataset of three mtDNA fragments and partitioned by genes. Each putative morphospecies is represented by a single terminal. The numbers above branches indicate bootstrap support values obtained in the IQ-TREE analysis using 5,000 UFboot iterations. The outgroups are omitted and the phylogenetic hypothesis for 388 ingroup terminals and 19 outgroups is shown in Fig. S1. The general appearance of specimens illustrates the multiple origins of similar aposematic patterns in distantly related species. Eight color patterns are defined in Methods. Photographs of all vouchers taken by the authors.

**Figure 3:**
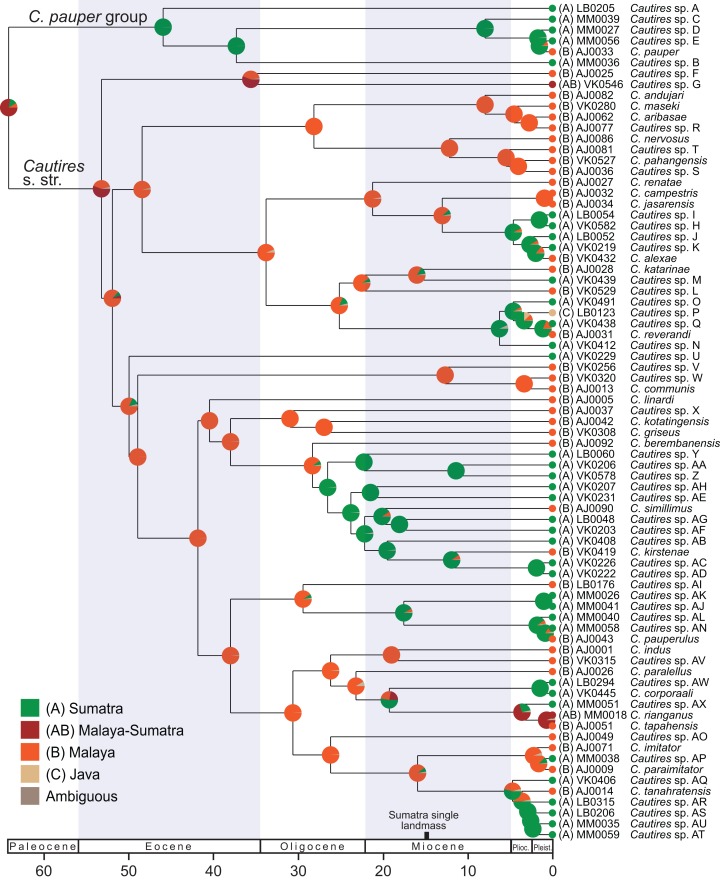
Ancestral distribution of *Cautires*. The reconstruction of the ancestral distribution of *Cautires* beetles in the Malay Peninsula and Sumatra inferred with the maximum likelihood framework implemented in BioGeoBEARS and using dataset containing 76 species. The tree shown was dated using the earlier estimated rates of molecular evolution of sequenced mitochondrial markers. The tRNA fragments were omitted from the analysis.

The dataset for subsequent analyses was pruned to a single representative of each putative species (Figs. 2 and 3). The reduced dataset contained 76 terminals and *Xylobanus* sp. as a single outgroup and was used for both dating and area reconstruction analyses. The splits were dated in Beast 1.8.1 using the fixed topology inferred from the analysis of the pruned dataset (Drummond et al., 2006; Suchard & Rambaut, 2009). The HKY model, Yule Process and Lognormal Uncorrelated Relaxed Clock, as proposed in the Beast 1.8.1 manual, were set in the Beast analysis after the application of the GTR+I+G model did not reach convergence (Drummond et al., 2012; Drummond & Bouckaert, 2015). As no fossils of Metriorrhynchini are available, we used rates of molecular evolution proposed by Papadopoulou, Anastasiou & Vogler (2010): the 0.0168 substitutions/site/my/lineage for *cox1* fragment, 0.0054 subs/s/my/l for *rrnL* fragment and 0.012 subs/s/my/l for *nad5* fragment. The Markov chain Monte Carlo (MCMC) parameters were set to 5 × 10^7^ million generations with sampling every 5,000 generations and the effective sample size values. The pre-stationary phase was identified in Tracer 1.6 (Rambaut et al., 2014) and the initial 1.25 × 10^7^ generations were discarded as burn-in.

Additionally, the ancestral areas were inferred using the ML framework in BioGeoBEARS (Matzke, 2013) implemented in RASP 4.0 (Yu et al., 2015), using the dataset containing 76 terminals (a single terminal per species). We compared all alternative models of colonization, all also with +J (Matzke, 2014) which tests founder-event speciation (Table S4). The localities were assigned to respective taxa and coded for two analyses. (i) general geographic origin (A) Sumatra and (B) Malaya; (ii) specific geographic origin: (A) Malay Highlands, (B) Malay lowlands and lower elevation forest <1,000 a. s. l., (C) Sumatra Jambi (Tujuh and Kerinci) (D) Sumatra Barat (Merapi, Maninjau, Talamau) and (E) Sumatra Utara (Sibayak and Sinabung) and (F) Java. The phylogenetic distribution of aposematic patterns was noted for each terminal and their geographic distribution was summarized on the map of the western part of the Sunda Shelf. We do not present the formal reconstruction of the evolution of color patterns because their distribution mostly agrees with specific geographic distribution (see the analysis above).

## Results

### Sanger sequencing, alignment, phylogenetic analyses

Three mtDNA fragments, *rrnL*, *cox1* and *nad5* were assembled in the dataset of 388 ingroup and 18 outgroup specimens. The ingroup was represented by 369 *cox1* fragments (95% completeness, 1,104 homologous positions in the MAFFT alignment), 178 *rrnL* (45%, 817 positions) and 368 *nad5* (95%, 1,322 positions) (Table S1). *Cautires* was retrieved as a monophyletic group albeit with moderate support BS 90%, similar to the relationships among the deepest clades (Figs. 2 and 3; Fig. S1). The shallower splits had high bootstrap (mostly BS ≥ 99%). A well-supported split separates the *Cautires pauper* species-group and the *Cautires* s. str. clade (i.e., all *Cautires* including the *C. obsoletus* species-group as defined by Dudkova & Bocak (2010), Jiruskova & Bocak (2015)).

### Species identification and distribution

Using morphology, we identified 76 species, 28 of them formally named, and the results of morphology-based species limits were compared with their genetic divergence (Fig. S1; Table S3). The delimitation widely agrees and in the cases of ambiguous support, the species are morphology-based using genitalia (e.g., *C. rianganus* and *C. tapahensis*) or the shape of the pronotum (e.g., *C. corporaali* and *Cautires* sp. AW; see illustrations in Fig. S1). Altogether 39 species were recorded on the Malay Peninsula and 39 species in Sumatra. Only two species (*Cautires* sp. G and *C. rianganus*) were simultaneously recorded in both landmasses. The highest local diversity was identified in the lower montane forests of both regions: the Cameron Highlands (24 spp.), the Sinabung and Sibayak volcanoes (10 spp.) and the Kerinci massif (22 spp.; Fig. 3, Inset (A)–(B)). About two-thirds of species were recorded only in a single locality.

Dispersal-extinction cladogenesis including founder-event speciation (DEC+J; Table S4) was identified as the most appropriate model of ancestral area reconstruction. Two deeply split clades, designated as *Cautires* s. str. and *C. pauper* group, split in the Paleocene (64.1 mya; Figs. 2 and 3; Fig. S2). The *C. pauper* group comprised six putative species; five of them from Sumatra and *C. pauper* from the lowlands of the Malay Peninsula (Fig. S2). *Cautires* s. str. started its diversification early and 20 splits were identified from 53 to 26 mya. One deeply rooted species occurs in Sumatra (*Cautires* sp. U), but as it is a single species, we cannot estimate its colonization history. Further, three Sumatran clades, representing 18 spp. in total, split from their Malay relatives at 26.5, 17.5 and 4.7 mya (Fig. 3; Figs. S2 and S3). Three Malay species were identified within these clades as single-species terminal lineages and their splits from the closest Sumatran relatives cannot be reliably dated (Fig. 3). We inferred 11 range shifts from Malaya to Sumatra, 10 range shifts in the opposite direction. Additionally, we inferred five transfers between the Malay lowlands and mountains (Fig. S3).

### Distribution of color patterns

**Figure 4:**
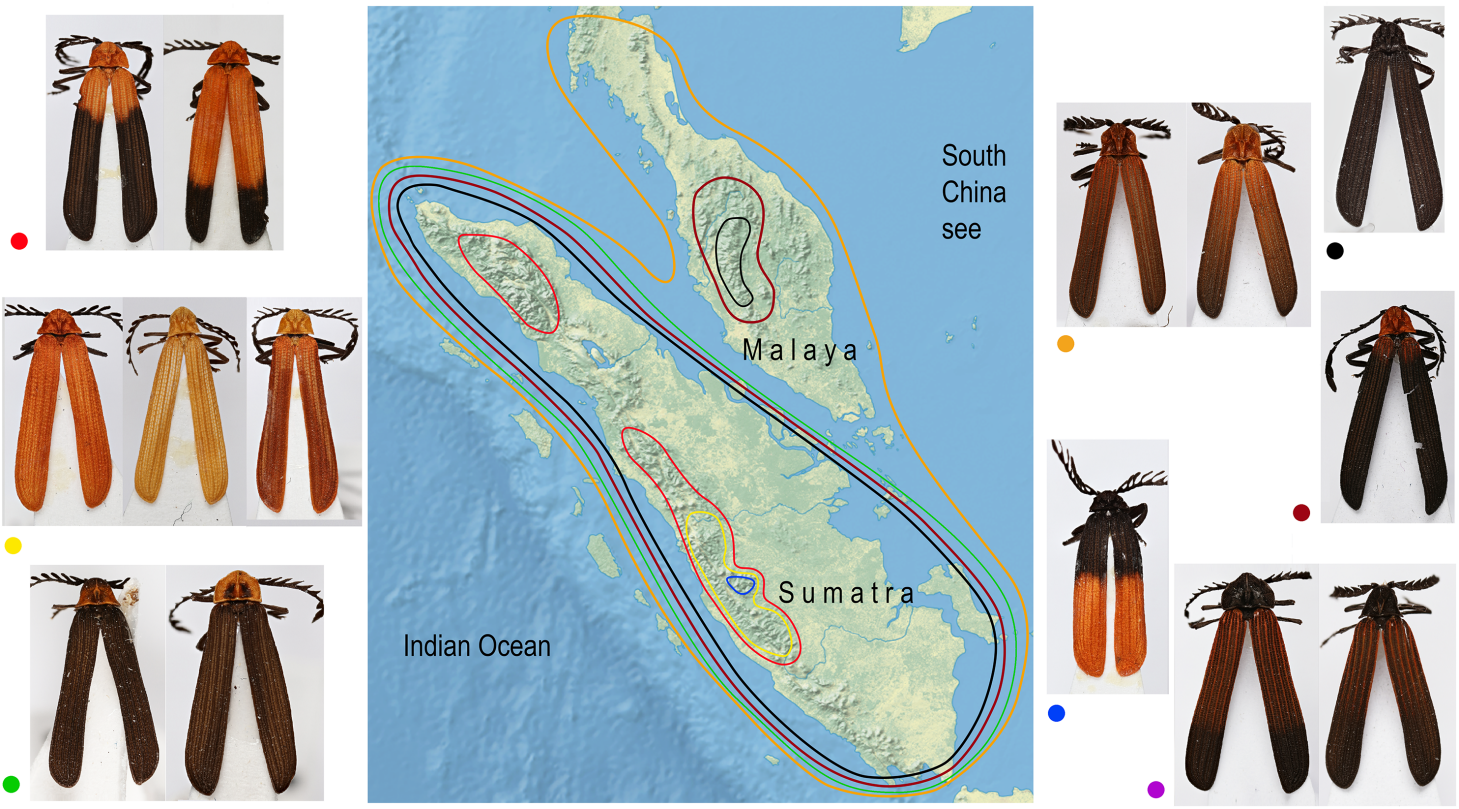
Distribution of aposematic patterns. The geographic distribution of aposematic patterns of *Cautires* in the Malay Peninsula and Sumatra. The list of species and color patterns from each locality shows the number of patterns and the observed alpha-diversity, the color codes are given at characteristic representatives of individual patterns and used in the map. All photographs of vouchers taken by the authors.

Similar coloration was identified in a high number of unrelated taxa (Fig. 2; Fig. S4; Table S5) and individual patterns occurred in restricted ranges: the lowland Malay *Cautires* were less brightly colored and had a brown to orange-brown pronotum and the humeral part of elytra with a gradual transition between bright and dark colored parts (Fig. 4). Most Malay montane species were uniformly black (13 spp.), some had a dark red colored pronotum and humeri (Fig. S5). The Sumatran low elevation species are uniformly black, have a red colored pronotum and black elytra, a black pronotum and the red basal part of elytra or they have a brown to orange-brown pronotum and the humeral part of the elytra (Fig. 4; Fig. S4). The Sumatran montane species are bright colored and they usually have a high-contrast border between dark and bright parts: 21 species are orange and black, further species are uniformly bright colored or they have the black pronotum and basal part of elytra in contrast with their bright red apical part. Distribution of all patterns is summarized in Fig. 4. The populations of a single species were generally uniform in color pattern and the observed differences were subtle. Seldom two patterns were found within a single population (observed in *Cautires* spp. AH, AX and T) or in two geographically distant populations (*C. rianganus* and *C. jasarensis*).

## Discussion

Altogether 76 putative species were defined based on morphological uniqueness and widely confirmed by the DNA divergence (Fig. 2; Fig. S1). The mitochondrial markers are known for incomplete lineage sorting and introgression and the species limits based on the divergence of mitochondrial DNA do not necessarily fit with the biological species defined using all evidence (Baselga et al., 2013). To handle this problem, we accepted as separate species only those sets of individuals and/or populations which differ in morphological diagnostic traits. Several terminal clades contain closely related, that is, recently diversified, species (Fig. 3) and a detailed study of their genomes might recover their complex origins as has been shown in other net-winged beetles (Bray & Bocak, 2016; Bocek & Bocak, 2016) or recently in *Heliconius* butterflies (Edelman et al., 2018). As we do not have genomic data, we prefer to delimit all species using morphology.

The above-described phylogeny and distribution of *Cautires* show that unrelated sympatrically occurring species resemble each other (Figs. 2 and 4). As a rule, net-winged beetles and their Batesian mimics, for example, moths, trues bugs and wasps, are highly similar in each locality (Linsley, Eisner & Klots, 1961; Eisner, Kafatos & Linsley, 1962; Eisner et al., 2008; Malohlava & Bocak, 2010; Bocak & Yagi, 2010; Lingafelter, 2013; Bocek & Bocak, 2016; Motyka, Kampova & Bocak, 2018). Here, we focus our discussion on the aposematic patterns of *Cautires*, because other lycids and lycid-like insects have not been included in the analysis. The color differences in *Cautires* do not necessarily indicate a separate species (Jiruskova & Bocak, 2015; Jiruskova, Motyka & Bocak, 2016) and we identified a few cases of intraspecific and intrapopulation color polymorphism indicated by the shared external morphology, the structure of genitalia and similar mtDNA sequences and variable color patterns as the only observed difference. The model of Müllerian mimicry does not predict multiple patterns, but polymorphism in warning colors is commonly encountered in nature (Sherratt, 2008; Motyka, Kampova & Bocak, 2018) and indicates the natural selection acting in concert with local community composition (Aubier & Sherratt, 2015).

A total of 18 localities across the Malay Peninsula and Sumatra were sampled to assess geographic genetic and phenotypic variation (Fig. 1). Although there are 76 spp. in the current analysis compared to 53 spp. formally described species from the region, we suppose that further species will be discovered in the future and that our sampling remains incomplete.

Despite these limitations, the data are sufficient to consider the relative age of the Malay and Sumatran fauna, species turnover between these regions, the number of colonization events across the Malacca Strait in the Neogene and the distribution of aposematic patterns.

### Origin of *Cautires* and their diversification

Deep-rooted Oriental *Cautires* lineages originated out of the studied region, namely in drifting India or in a contact zone between India and continental Asia at the time of their collision (55–35 mya, Sklenarova, Chesters & Bocak, 2013). Additionally, synonymous nucleotide divergence and saturation limit the robustness of deep mtDNA-based topologies. Therefore, no splits beyond ∼30 mya are considered (Fig. 3; Fig. S3). Fossil and tectonic calibrations are unavailable and the secondary calibration would be ambiguous due to sparse sampling and large differences between various analyses (Hunt et al., 2007; McKenna et al., 2015; Toussaint et al., 2017a; Bocak et al., 2016; Zhang et al., 2018; Kusy et al., 2018). Therefore, we used the rates of mitogenome evolution and discussed only Late Paleogene and Neogene splits. Our rate-based dating is supported by the congruence of the evolution of the Sumatran *Cautires* fauna and tectonics of Sumatra (Fig. 3).

We suppose that *Cautires* started their diversification in the region in the Oligocene (Fig. 3). Until the Lower Pleistocene, the *C. pauper* group contained only Sumatran species and only 1.5 mya a single species colonized Malaya (Figs. 2 and 3). *Cautires* s. str. is a lineage of Malay origins and almost all ancestral lineages only occurred on the Malay Peninsula or Asian continent, respectively, prior to the Upper Oligocene (26.5 mya; Fig. 3). The Sumatran fauna consists mostly of terminal subclades nested in older, more inclusive Malay groups similarly to *Platerodrilus* Pic, 1921 net-winged beetles (Masek et al., 2015). The oldest Sumatran highly diverse clade split from Malay relatives in the Upper Oligocene (10 Sumatran spp. and two Malay spp. in terminal positions; Fig. 3). Further splits between Malay and Sumatran lineages are dated to the Miocene (17.5 and 6.2 mya) and later in the Pliocene (three events, 4.7, 4.6 and 3.6 mya). Hence, the Sumatran *Cautires* diversified with an apparent delay compared to the Malay fauna in accord with the tectonic history (Fig. 3; Hall, 2002). The origin of the oldest Sumatran clade supports the existence of an island chain in the region from the Upper Oligocene to the Lower Miocene (Hall, 2002; Malohlava & Bocak, 2010; Masek et al., 2015). The colonization direction was asymmetrical from the Upper Oligocene until the end of the Upper Pliocene. In this period, we identified six colonization events from the Malay Peninsula to Sumatra which gave origin to multi-species clades, and no colonization in the opposite direction (Fig. 4). This pattern agrees with the hypothesis that areas with recent histories of size expansion should obtain higher levels of immigration (De Bruyn et al., 2014) and it was earlier documented in net-winged beetle *Scarelus* Waterhouse, 1879 and *Platerodrilus* (Malohlava & Bocak, 2010; Masek et al., 2015). The phylogeny and reconstruction of ancestral areas suggest a founder effect diversification model and subsequent in situ speciation producing different faunas of neighboring landmasses (Figs. 3 and 4; Matzke, 2014; Demos et al., 2016).

We identified colonization events in both directions during the Quaternary and this is in line with repeated low sea levels (Voris, 2000). We propose that colonization dynamics shifted from a tectonic to a climatic dominated regime in the last 5 million years. Aside from two species recorded from both regions, all colonization events resulted in the origin of a separate species or a whole local clade (Fig. 3). These colonization events contributed to the observed species-level diversity (Fig. 2). Although the sampling is apparently incomplete, we can conclude that most *Cautires* have small ranges and that the geographic speciation mode of speciation is frequent between Sumatra and the Malay Peninsula (Barraclough & Vogler, 2000; Ikeda, Nishikawa & Sota, 2012). We suppose that intensive faunal exchange between these regions would result in the presence of widespread species on both sides of the Malacca Strait. Our data do not support such a prediction.

Further aspects of the colonization and diversification history are the origin and uniqueness of the montane faunas. We identified 19 *Cautires* with distribution limited to the montane forests in the Malay Central Range and none of them is distributed in a wide range of elevations (Table S1; Jiruskova, Motyka & Bocak, 2016). Two clades of the Malayan montane fauna started their in situ diversification 28.1 and 12.6 mya and they represent about a half of the diversity reported from Malay mountains. Despite the limited extent of mountain regions on the Malay Peninsula and a turbulent climatic history which could have potentially caused complex altitudinal range shifts over such long periods, these two clades are dominantly mountainous and only a single species, *Cautires* sp. S, was inferred to be a member of the mountain clade yet was distributed in lower elevations (Fig. 4; Fig. S1). Additional nine species were recorded in the Malay mountains and the time of their split from lowland relatives cannot be exactly estimated. The Malay Central Range is a biodiversity hotspot with ancient and diverse fauna similar to other tropical mountains (Merckx et al., 2015). Similarly to the Malay Peninsula, we found a high turnover between Sumatran mountains and lowlands. Only three species were recorded simultaneously in two mountain regions of Sumatra—*Cautires* spp. B, N and AN. We conclude that the in situ diversification of montane species contributed to high alpha-taxonomic diversity also in Sumatra (Merckx et al., 2015; Demos et al., 2016).

To avoid a potential source of error when closely related species are delimited, we can alternatively consider the phylogeny of *Cautires* only from the Rupelian Stage of the Oligocene (∼30 mya) to the beginning of the Pliocene (∼5 mya). *Cautires* s. str. already contained 17 separate lineages at the beginning of the Oligocene, all (with one exception) known only from the Malay Peninsula (*Cautires* sp. G, see Fig. 3). Before Sumatra was uplifted, some of these lineages dispersed to proto-Sumatran islands and diversified there. Further colonization events are hypothesized from the Malay Peninsula to Sumatra in the Upper Miocene and the Lower Pliocene, about 5–7 mya (Fig. 3). At the beginning of the Pliocene, the *Cautires* s. str. hypothetically contained 45 lineages, 17 of them Sumatran (Fig. 4). The data suggest that despite geographic proximity, colonization events had been rare in the region for a long time and unique Malayan and Sumatran faunas were established already before the Pliocene/Pleistocene period.

### Why does the Malacca Strait separate different faunas?

The Malacca Strait is shallow and, especially in the southern part, very narrow, so it should not represent a major dispersal barrier for flying insects (Fig. 1; Balke et al., 2009; Taänzler et al., 2014; Toussaint et al., 2017b). Additionally, very similar ecosystems are currently present on the Malay Peninsula and Sumatra and we suppose that the narrow Malacca Strait never separated ecosystems whose differences could substantially lower the colonization success (Thomas, 1994; Morley, 2000). We cannot exclude a possibility that some species might colonize other islands on the Sunda Shelf first and only then colonize Sumatra or Malaya, respectively. The distinct fauna of Borneo (Sklenarova, Chesters & Bocak, 2013; Masek et al., 2018) indicates that colonization via Borneo did not dominate and can only marginally affect the species-level structure of both studied faunas. Observed small ranges point to low colonization capacity which makes the shortest distance colonization direction the most probable (Lester et al., 2007; Bray & Bocak, 2016).

Hence, we can discuss further factors which might be responsible for the observed distribution. The reconstruction of the paleoclimate during recent glacial maxima indicates that the Malacca Strait was covered by semi-dry savannah (Cannon, Morley & Bush, 2009; DiNezio & Tierney, 2013). We identified a lower abundance and diversity of *Cautires* in lowland localities characterized by a more pronounced dry season than in mountain ecosystems. The ecosystems of the exposed Shelf during glacial maxima were unfavorable (Gathorne-Hardy et al., 2002) and although more colonization events were recovered since the Pliocene, most species are endemic to the respective area.

Factors hypothetically decreasing the colonization potential are different aposematic color patterns in the lowlands and individual mountain massifs of the Malay Peninsula and Sumatra. Similarly-colored *Cautires* are unrelated (Fig. 2) and the geographic distribution of various aposematic patterns is limited (Fig. 4). Based on these facts, we can hypothesize that dispersing *Cautires* regularly entered the area where their aposematic signal was uncommon or absent. Therefore, we propose that some dispersing populations could be wiped out by local predators unfamiliar with their allochthonous aposematic signal before they could adapt to local mimetic complexes, that is, they could be under antiapostatic selection decreasing colonization rates (Beatty, Beirinckx & Sherratt, 2004; Sherratt, 2008).

## Conclusions

The current analysis of *Cautires* morphology and the mtDNA dataset indicates that different *Cautires* faunas are separated by the shallow and commonly dry Malacca Strait and that the independent in situ speciation in respective areas is characteristic in the Malay Peninsula and Sumatra since the Oligocene. The faunas have a high level of endemism and a different diversification history. Most deeply-rooted lineages evolved on the Malay Peninsula and some of them colonized Sumatra where they subsequently diversified. Surprisingly, colonization events were uncommon despite the close geographic position and similar ecosystems. Even the relatively recent colonization events across the Malacca Strait in the Upper Pliocene and Pleistocene were followed by speciation. The species colonizing a new range adopted local mimetic patterns and we suppose that the selection against rare aposematic patterns limits the colonization capacity of unpalatable *Cautires*, but simultaneously it may enhance speciation (Bocak & Yagi, 2010; Bray & Bocak, 2016). The Malay montane fauna is of ancient origin, contains a high proportion of endemic species and represents a biodiversity island. The survival of South East Asian fauna is under ever-increasing human pressure (Sodhi et al., 2004) and we demonstrate that the Malay fauna contains all deep lineages; that is, it has higher phylogenetic diversity and therefore has a much higher value for conservation if priorities are to be set in this region (Lawing & Matzke, 2014).

## Supplemental Information

10.7717/peerj.6511/supp-1 Supplemental Information 1 Supplementary Information. The list of studies material, dataset characteristics, models, genetic distances, aposematic patterns, dated phylogenetic tree, reconstruction of ancestral distribution and aposematic patterns.

10.7717/peerj.6511/supp-2 Supplemental Information 2 Molecular dataset. The aligned data for 406 taxa and three mitochondrial markers.

## Additional Information and Declarations

### Field Study Permissions

The following information was supplied relating to field study approvals (i.e., approving body and any reference numbers): Field collecting in Malaysia was permitted by Permit Penyelidikan No TS/PTD/5/4Jld48(41) by Lembaga Pemegang Amanah Taman-Taman. Other material was collected on public land outside protected areas and species included in the research are not protected by international or national laws.

### DNA Deposition

The following information was supplied regarding the deposition of DNA sequences: The sequences are available in GenBank: MK467618–MK468468.

### Data Availability

The following information was supplied regarding data availability: The raw data are available in a Supplemental File.
