# Supplementary material for: The Malacca Strait separates distinct faunas of poorly-flying Cautires net-winged beetles
Source: PeerJ. 2019 Mar 5;7:e6511. doi: 10.7717/peerj.6511 (PMC6407506; doi:10.7717/peerj.6511)
Supplement: Supplemental Information 1 — The list of studies material, dataset characteristics, models, genetic distances, aposematic patterns, dated phylogenetic tree, reconstruction of ancestral distribution and aposematic patterns. [file peerj-07-6511-s001.pdf]

Does the Malacca Strait separate distinct faunas of poorly flying Cautires net-winged beetles?

Alice Jiruskova, Michal Motyka, Matej Bocek and Ladislav Bocak*

Laboratory of Molecular Systematics, Department of Zoology, Faculty of Science, Palacky University, 17. listopadu 50, 771 46 Olomouc, Czech Republic
*corresponding author: ladislav.bocak@upol.cz

List of supplementary material.

Supplementary Table S1. The list of sequenced specimens with geographic origin and GenBank Accession Numbers.
Supplementary Table S2. Characteristics of concatenated super-matrices: partition schemes and used models of DNA evolution.
Supplementary Table S3. Uncorrected genetic distances in *Cautires cox1* mitochondrial DNA fragment.
Supplementary Table S4. Log likelihood and Akaike information criterion (AIC) values for every model compared in the BIOGEOBEARS supermodel using RASP 4.0.
Supplementary Table S5. The list of sequenced ingroup terminals with description of dorsal colour patterns.
Supplementary Figure S1. Maximum likelihood analysis of *Cautires* dataset including outgroup using IQ-TREE. Numbers on branches represent ultrafast bootstrap values.
Supplementary Figure S2. Dated phylogenetic tree of *Cautires* using BEAST with constrained topology inferred from the maximum likelihood analysis of the three-gene dataset.
Supplementary Figure S3. Time calibrated maximum clade credibility tree of *Cautires* with reconstruction of specific geographic localities.
Supplementary Figure S4. Aposematic patterns of *Cautires* net-winged beetles in the Malay Peninsula and Sumatra.

Supplementary table S1. The list of sequenced specimens with geographic origin and GenBank Accession Numbers.

Voucher number	Taxon	Locality	mitochondrial DNA/Acc. Number		
			cox1	*rrnL*	*nad5*
**Outgroup**					
VK0158	*Xylobanus* sp.	Perak, Ringlet, 40 km SE of Ipoh, 900 m	AB123456	AB123456	AB123456
VK0067	*Xylobanus* sp.	Pahang, rd. Ipoh–Kg. Raja, 35 km Ipoh, 800 m	AB123456	AB123456	AB123456
AJ0023	*Xylobanus* sp.	Pahang, Kg. Kuala Boh, 960 m	KR140287	AB123456	AB123456
VK0175	*Xylobanus* sp.	Sumatra Utara, Brastagi, Gn. Sibayak 1800 m	AB123456	AB123456	AB123456
000121	*Metanoeus* sp.	Sumatra	KC538277	KC538662	KC538468
000372	*Porr. rhipidium*	Australia	DQ144678	KC538699	DQ144702
000378	*P. haemorrhoidalis*	Australia	DQ144679	KC538704	DQ144703
000199	*Microtrichalus* sp.	Sulawesi	KC538290	KC538674	KC538481
000376	*Microtrichalus* sp.	Australia	KC53816	KC538703	KC538507
MW0008	*Metanoeus* sp.	Jambi, Gn. Kerinci	AB123456	AB123456	AB123456
000105	*Metanoeus* sp.	Borneo	KC538271	KC538657	KC538463
000248	*Metanoeus* sp.	Sumatra	KC538299	KC538684	KC538490
MW0021	*Metanoeus* sp.	Sumatra Utara, Brastagi, Gn. Sibayak	AB123456	AB123456	AB123456
MW0025	*Metanoeus* sp.	Sumatra Utara, Brastagi, Gn. Sibayak	AB123456	AB123456	AB123456
LB0484	*Leptotrichalus* sp.	Sumatra Barat, Gn. Merapi	AB123456	–	AB123456
LB0131	*Diatrichalus* sp.	Sumatra Barat, Gn. Merapi	AB123456	AB123456	AB123456
LB0514	*Microtrichalus* sp.	Sumatra Barat, Gn. Merapi	AB123456	–	AB123456
LB0196	*Microtrichalus* sp.	Pahang, 30 km NE of Raub	AB123456	–	AB123456
**Ingroup**					
VK0432	*Cautires alexae*	Perak, 40 km SE of Ipoh, 900 m, Ringlet	AB123456	–	–
VK0433	*C. alexae*	Perak, 40 km SE of Ipoh, 900 m, Ringlet	AB123456	–	–
VK0426	*C. andujari*	Pahang, Tanah Rata, Gn. Jasar, 1600 m	AB123456	AB123456	AB123456
AJ0082	*C. andujari*	Pahang, Brinchang, Gn. Brinchang, 1800 m	AB123456	AB123456	AB123456
AJ0083	*C. andujari*	Pahang, Brinchang, Gn. Brinchang, 1800 m	AB123456	AB123456	AB123456
VK0251	*C. andujari*	Pahang, Tanah Rata, Gn. Jasar, 1600 m	AB123456	–	AB123456
VK0272	*C. andujari*	Pahang, Tanah Rata, Gn. Jasar, 1500–1800 m	AB123456	–	AB123456
VK0312	*C. andujari*	Pahang, Tanah Rata, Gn. Jasar, 1500–1800 m	AB123456	–	AB123456
VK0322	*C. andujari*	Pahang, Tanah Rata, Gn. Jasar, 1500–1800 m	AB123456	–	AB123456
VK0519	*C. andujari*	Pahang, Tanah Rata, Gn. Jasar, 1500–1800 m	AB123456	AB123456	AB123456
VK0564	*C. andujari*	Pahang, Tanah Rata, Gn. Jasar, 1600 m	AB123456	–	AB123456
AJ0058	*C. arribasae*	Pahang, Gn. Beremban	AB123456	AB123456	AB123456
AJ0059	*C. arribasae*	Pahang, Gn. Beremban	AB123456	AB123456	AB123456
AJ0062	*C. arribasae*	Pahang, Gn. Beremban	AB123456	AB123456	AB123456
AJ0064	*C. arribasae*	Pahang, Gn. Beremban	AB123456	AB123456	AB123456
AJ0066	*C. arribasae*	Pahang, Gn. Beremban	AB123456	–	AB123456
AJ0078	*C. arribasae*	Pahang, Gn. Beremban	AB123456	AB123456	–
AJ0079	*C. arribasae*	Pahang, Gn. Beremban	AB123456	AB123456	AB123456
AJ0092	*C. berembanensis*	Pahang, Gn. Beremban, 1480 m	KR140290	AB123456	AB123456
VK0254	*C. berembanensis*	Pahang, Tanah Rata, Gn. Jasar, 1500–1800 m	AB123456	AB123456	AB123456
VK0275	*C. berembanensis*	Pahang, Tanah Rata, Gn. Jasar, 1500–1800 m	AB123456	–	AB123456
VK0278	*C. berembanensis*	Pahang, Tanah Rata, Gn. Jasar, 1500–1800 m	AB123456	–	AB123456
AJ0096	*C. campestris*	Pahang, 12 km, rd. Kg. Raja–Gua Musang, 980 m	–	AB123456	AB123456
AJ0032	*C. campestris*	Pahang, 12 km, rd. Kg. Raja–Gua Musang, 980 m	AB123456	AB123456	AB123456
AJ0039	*C. campestris*	Perak, 24 km, rd. Tapah–Ringlet, 350 m	AB123456	AB123456	AB123456
AJ0040	*C. campestris*	Perak, 24 km, rd. Tapah–Ringlet, 350 m	AB123456	AB123456	AB123456
AJ0041	*C. campestris*	Perak, 24 km, rd. Tapah–Ringlet, 350 m	AB123456	AB123456	AB123456
AJ0004	*C. communis*	Pahang, Gn. Jasar, N slope, 1550 m	AB123456	AB123456	–
AJ0006	*C. communis*	Pahang, Gn. Jasar, N slope, 1550 m	AB123456	AB123456	–
AJ0013	*C. communis*	Pahang, Gn. Beremban, 1480 m	AB123456	AB123456	AB123456
AJ0061	*C. communis*	Pahang, Gn. Beremban, 1480 m	AB123456	AB123456	AB123456
AJ0093	*C. communis*	Pahang, Gn. Beremban, 1480 m	AB123456	AB123456	AB123456
AJ0068	*C. communis*	Pahang, Gn. Beremban, 1480 m	AB123456	AB123456	AB123456
AJ0084	*C. communis*	Pahang, Brinchang, Gn. Brinchang, 1800 m	AB123456	AB123456	AB123456
AJ0087	*C. communis*	Pahang, Brinchang, Gn. Brinchang, 1800 m	AB123456	AB123456	AB123456
AJ0091	*C. communis*	Pahang, Gn. Beremban, 1480 m	AB123456	AB123456	AB123456
VK0242	*C. communis*	Pahang, Tanah Rata, Gn. Jasar, 1500–1800 m	AB123456	–	AB123456
VK0244	*C. communis*	Pahang, Tanah Rata, Gn. Jasar, 1500–1800 m	AB123456	–	AB123456
VK0245	*C. communis*	Pahang, Tanah Rata, Gn. Jasar, 1500–1800 m	AB123456	–	AB123456
VK0246	*C. communis*	Pahang, Tanah Rata, Gn. Jasar, 1500–1800 m	AB123456	–	AB123456
VK0255	*C. communis*	Pahang, Tanah Rata, Gn. Jasar, 1500–1800 m	AB123456	–	AB123456
VK0260	*C. communis*	Pahang, Tanah Rata, Gn. Jasar, 1500–1800 m	AB123456	AB123456	AB123456
VK0261	*C. communis*	Pahang, Tanah Rata, Gn. Jasar, 1500–1800 m	AB123456	AB123456	AB123456
VK0262	*C. communis*	Pahang, Tanah Rata, Gn. Jasar, 1500–1800 m	AB123456	–	AB123456
VK0263	*C. communis*	Pahang, Tanah Rata, Gn. Jasar, 1500–1800 m	AB123456	–	AB123456
VK0264	*C. communis*	Pahang, Tanah Rata, Gn. Jasar, 1500–1800 m	AB123456	–	AB123456
VK0265	*C. communis*	Pahang, Tanah Rata, Gn. Jasar, 1500–1800 m	AB123456	–	AB123456
VK0267	*C. communis*	Pahang, Tanah Rata, Gn. Jasar, 1500–1800 m	AB123456	–	AB123456
VK0271	*C. communis*	Pahang, Tanah Rata, Gn. Jasar, 1500–1800 m	AB123456	–	AB123456
VK0274	*C. communis*	Pahang, Tanah Rata, Gn. Jasar, 1500–1800 m	AB123456	–	AB123456

VK0276	*C. communis*	Pahang, Tanah Rata, Gn. Jasar, 1500–1800 m	AB123456	–	AB123456
VK0277	*C. communis*	Pahang, Tanah Rata, Gn. Jasar, 1500–1800 m	AB123456	–	AB123456
VK0279	*C. communis*	Pahang, Tanah Rata, Gn. Jasar, 1500–1800 m	AB123456	–	AB123456
VK0310	*C. communis*	Pahang, Tanah Rata, 1400–1650 m	AB123456	–	AB123456
VK0311	*C. communis*	Pahang, Tanah Rata, Gn. Jasar, 1500–1800 m	AB123456	–	AB123456
VK0313	*C. communis*	Pahang, Tanah Rata, Gn. Jasar, 1500–1800 m	AB123456	–	AB123456
VK0314	*C. communis*	Pahang, Tanah Rata, Gn. Jasar, 1500–1800 m	AB123456	–	AB123456
VK0316	*C. communis*	Pahang, Tanah Rata, Gn. Jasar, 1500–1800 m	AB123456	–	AB123456
VK0317	*C. communis*	Pahang, Tanah Rata, Gn. Jasar, 1500–1800 m	AB123456	–	AB123456
VK0321	*C. communis*	Pahang, Tanah Rata, Gn. Jasar, 1500–1800 m	AB123456	–	AB123456
VK0323	*C. communis*	Pahang, Tanah Rata, Gn. Jasar, 1500–1800 m	AB123456	–	AB123456
VK0324	*C. communis*	Pahang, Tanah Rata, Gn. Jasar, 1500–1800 m	AB123456	–	AB123456
VK0325	*C. communis*	Pahang, Tanah Rata, Gn. Jasar, 1500–1800 m	AB123456	–	AB123456
VK0512	*C. communis*	Pahang, Tanah Rata, Gn. Jasar, 1500–1800 m	AB123456	–	AB123456
VK0513	*C. communis*	Pahang, Tanah Rata, Gn. Jasar, 1500–1800 m	AB123456	–	AB123456
VK0514	*C. communis*	Pahang, Tanah Rata, Gn. Jasar, 1500–1800 m	AB123456	–	AB123456
VK0515	*C. communis*	Pahang, Tanah Rata, Gn. Jasar, 1500–1800 m	AB123456	–	AB123456
VK0520	*C. communis*	Pahang, Tanah Rata, Gn. Jasar, 1500–1800 m	AB123456	–	AB123456
VK0521	*C. communis.*	Pahang, Tanah Rata, Gn. Jasar, 1500–1800 m	AB123456	–	AB123456
VK0523	*C. communis*	Pahang, Tanah Rata, Gn. Jasar, 1500–1800 m	AB123456	–	AB123456
VK0524	*C. communis*	Pahang, Tanah Rata, Gn. Jasar, 1500–1800 m	AB123456	–	AB123456
VK0421	*C. communis*	Pahang, Tanah Rata, 1600 m	AB123456	–	AB123456
VK0361	*C. communis*	Pahang, Tanah Rata, 1600 m	AB123456	–	AB123456
VK0572	*C. communis*	Pahang, Tanah Rata, 1600 m	AB123456	–	AB123456
VK0526	*C. communis*	Pahang, Tanah Rata, 1600 m	AB123456	–	AB123456
VK0418	*C. communis*	Pahang, Tanah Rata, 1600 m	AB123456	–	AB123456
VK0268	*C. communis*	Pahang, Tanah Rata	AB123456	–	AB123456
VK0450	*C. communis*	Pahang, Tanah Rata	AB123456	–	AB123456
VK0563	*C. communis*	Pahang, Tanah Rata, Gn. Jasar, 1600 m	AB123456	–	AB123456
VK0568	*C. communis*	Pahang, Tanah Rata, Gn. Jasar, 1600 m	AB123456	–	AB123456
VK0619	*C. communis*	Pahang, Tanah Rata, Gn. Jasar, 1600 m	AB123456	–	AB123456
VK0622	*C. communis*	Pahang, Tanah Rata, Gn. Jasar, 1600 m	AB123456	–	AB123456
VK0623	*C. communis*	Pahang, Tanah Rata, Gn. Jasar, 1600 m	AB123456	–	AB123456
MM0054	*C. corporaali*	Sumatra Utara, Brastagi, Gn. Sibayak	AB123456	–	AB123456
MM0057	*C. corporaali*	Sumatra Utara, Brastagi	AB123456	–	–
VK0445	*C. corporaali*	Sumatra Utara, Brastagi, Gn. Sibayak, 1800m	AB123456	AB123456	AB123456
VK0308	*C. griseus*	Pahang, rd. Ipoh–Kg. Raja, 35 km E of Ipoh, 800 m	AB123456	AB123456	AB123456
VK0575	*C. griseus*	Pahang, Tanah Rata, 1600 m	AB123456	–	AB123456
AJ0071	*C. imitator*	Pahang, Gn. Beremban, 1480 m	KR140298	AB123456	AB123456
AJ0072	*C. imitator*	Pahang, Gn. Beremban, 1480 m	KR140299	AB123456	AB123456
MM0009	*C. imitator*	Cameron Highlands, 1600 m	KR140323	–	AB123456
AJ0001	*C. indus*	Perak, 40 km, rd. Tapah–Ringlet, 1030 m	KR140314	AB123456	AB123456
AJ0002	*C. indus*	Perak, 40 km, rd. Tapah–Ringlet, 1030 m	KR140315	AB123456	AB123456
AJ0003	*C. indus*	Perak, 40 km, rd. Tapah–Ringlet, 1030 m	KR140316	AB123456	AB123456
AJ0007	*C. indus*	Pahang, Tioman, Kg. Tekek–Juara, 50–300 m	KR140318	AB123456	AB123456
AJ0015	*C. indus*	Perak, 47 km, Rd. Ipoh–Kg. Raja, 1030 m	KR140317	AB123456	AB123456
AJ0045	*C. indus*	Perak, 24 km, rd. Tapah–Ringlet, 350 m	KR140293	AB123456	AB123456
AJ0046	*C. indus*	Perak, 24 km, rd. Tapah–Ringlet, 350 m	KR140294	AB123456	AB123456
AJ0047	*C. indus*	Perak, 24 km, rd. Tapah–Ringlet, 350 m	KR140295	AB123456	AB123456
AJ0052	*C. indus*	Perak, 24 km, rd. Tapah–Ringlet, 350 m	KR140307	AB123456	AB123456
AJ0050	*C. indus*	Perak, 24 km, rd. Tapah–Ringlet, 350 m	KR140305	AB123456	AB123456
AJ0054	*C. indus*	Perak, 24 km, rd. Tapah–Ringlet, 350 m	KR140309	AB123456	AB123456
AJ0056	*C. indus*	Perak, 24 km, rd. Tapah–Ringlet, 350 m	KR140311	AB123456	AB123456
AJ0057	*C. indus*	Perak, 24 km, rd. Tapah–Ringlet, 350 m	KR140312	AB123456	AB123456
AJ0038	*C. indus*	Perak, 24 km, rd. Tapah–Ringlet, 350 m	KR140313	AB123456	AB123456
AJ0094	*C. indus*	Pahang, 12 km, rd. Kg. Raja–Gua Musang, 980 m	KR140319	AB123456	AB123456
MM0007	*C. indus*	Perak, 40 km SE of Ipoh	KR140322	–	AB123456
AJ0034	*C. jasarensis*	Pahang, 12 km, rd. Kg. Raja–Gua Musang, 980 m	AB123456	AB123456	AB123456
AJ0067	*C. jasarensis*	Pahang, Gn. Beremban, 1480 m	AB123456	AB123456	AB123456
AJ0070	*C. jasarensis*	Pahang, Gn. Beremban, 1480 m	AB123456	AB123456	AB123456
VK0252	*C. jasarensis*	Pahang, Tanah Rata, Gn. Jasar, 1600 m	AB123456	–	AB123456
VK0253	*C. jasarensis*	Pahang, Tanah Rata, Gn. Jasar, 1600 m	AB123456	AB123456	AB123456
VK0362	*C. jasarensis*	Pahang, Tanah Rata, Gn. Jasar, 1600 m	AB123456	–	AB123456
VK0569	*C. jasarensis*	Pahang, Tanah Rata, Gn. Jasar, 1600 m	AB123456	–	AB123456
VK0425	*C. jasarensis*	Pahang, Tanah Rata, Gn. Jasar, 1600 m	AB123456	–	AB123456
VK0437	*C. jasarensis*	Pahang, rd. Ipoh–Kg. Raja, 51 km E of Ipoh, 1100 m	AB123456	–	AB123456
VK0565	*C. jasarensis*	Pahang, Tanah Rata, Gn. Jasar, 1600 m	AB123456	–	AB123456
AJ0028	*C. katarinae*	Pahang, km 12, rd. Kg. Raja–Gua Musang, 980 m	AB123456	AB123456	AB123456
VK0419	*C. kirstenae*	Pahang, Tanah Rata, 1600 m	AB123456	AB123456	AB123456
AJ0042	*C. kotatinggensis*	Perak, 24 km, rd. Tapah–Ringlet, 350 m	AB123456	AB123456	AB123456
AJ0048	*C. kotatinggensis*	Perak, 24 km, rd. Tapah–Ringlet, 350 m	AB123456	AB123456	AB123456
A00048	*C. kotatinggensis*	Johor, 30 km N of Kotatinggi	HQ456967	HQ456948	HQ456990
A00050	*C. kotatinggensis*	Johor, 30 km N of Kotatinggi	AB123456	–	KC538546
AJ0005	*C. linardi*	Pahang, Gn. Jasar, N slope, 1550 m	AB123456	AB123456	AB123456
VK0451	*C. linardi*	Pahang, Tanah Rata, Gn. Jasar	AB123456	AB123456	AB123456
VK0625	*C. linardi*	Pahang, Tanah Rata, Gn. Jasar	AB123456	–	AB123456
AJ0069	*C. maseki*	Pahang, Gn. Beremban, 1480 m	AB123456	AB123456	AB123456

VK0280	*C. maseki*	Pahang, Tanah Rata, Gn. Jasar, 1500–1800 m	AB123456	AB123456	AB123456
VK0628	*C. maseki*	Pahang, Tanah Rata, Gn. Jasar, 1600 m	AB123456	–	AB123456
VK0576	*C. nervosus*	Pahang, Tanah Rata, Gn. Jasar, 1600 m	AB123456	–	AB123456
VK0573	*C. nervosus*	Pahang, Tanah Rata, Gn. Jasar, 1600 m	AB123456	–	AB123456
VK0570	*C. nervosus*	Pahang, Tanah Rata, Gn. Jasar, 1600 m	AB123456	AB123456	AB123456
VK0571	*C. nervosus*	Pahang, Tanah Rata, Gn. Jasar, 1600 m	AB123456	–	AB123456
VK0360	*C. nervosus*	Pahang, Tanah Rata, Gn. Jasar, 1600 m	AB123456	–	AB123456
VK0422	*C. nervosus*	Pahang, Tanah Rata, Gn. Jasar, 1600 m	AB123456	–	AB123456
VK0533	*C. nervosus*	Pahang, Tanah Rata, Gn. Jasar, 1600 m	AB123456	AB123456	AB123456
AJ0076	*C. nervosus*	Pahang, Gn. Jasar, E slope, 1500 m	AB123456	AB123456	–
VK0243	*C. nervosus*	Pahang, Tanah Rata, Gn. Jasar, 1500–1800 m	AB123456	–	AB123456
VK0257	*C. nervosus*	Pahang, Tanah Rata, Gn. Jasar, 1500–1800 m	AB123456	–	AB123456
VK0258	*C. nervosus*	Pahang, Tanah Rata, Gn. Jasar, 1500–1800 m	AB123456	–	AB123456
VK0266	*C. nervosus*	Pahang, Tanah Rata, Gn. Jasar, 1500–1800 m	AB123456	–	AB123456
VK0516	*C. nervosus*	Pahang, Tanah Rata, Gn. Jasar, 1500–1800 m	AB123456	–	AB123456
VK0517	*C. nervosus*	Pahang, Tanah Rata, Gn. Jasar, 1500–1800 m	AB123456	–	AB123456
VK0518	*C. nervosus*	Pahang, Tanah Rata, Gn. Jasar, 1500–1800 m	AB123456	–	AB123456
VK0525	*C. nervosus*	Pahang, Tanah Rata, Gn. Jasar, 1500–1800 m	AB123456	–	AB123456
VK0241	*C. nervosus*	Pahang, Tanah Rata, Gn. Jasar, 1500–1800 m	AB123456	–	AB123456
VK0273	*C. nervosus*	Pahang, Tanah Rata, Gn. Jasar, 1500–1800 m	AB123456	–	AB123456
VK0281	*C. nervosus*	Pahang, Tanah Rata, Gn. Jasar, 1500–1800 m	AB123456	–	AB123456
VK0282	*C. nervosus*	Pahang, Tanah Rata, Gn. Jasar, 1500–1800 m	AB123456	–	AB123456
VK0318	*C. nervosus*	Pahang, Tanah Rata, Gn. Jasar, 1500–1800 m	AB123456	–	AB123456
VK0319	*C. nervosus*	Pahang, Tanah Rata, Gn. Jasar, 1500–1800 m	AB123456	–	AB123456
VK0621	*C. nervosus*	Pahang, Tanah Rata, Gn. Jasar, 1600 m	AB123456	–	AB123456
VK0618	*C. nervosus*	Pahang, Tanah Rata, Gn. Jasar, 1600 m	AB123456	–	AB123456
VK0453	*C. nervosus*	Pahang, Tanah Rata, Gn. Jasar, 1600 m	AB123456	–	AB123456
VK0269	*C. nervosus*	Pahang, Tanah Rata, Gn. Jasar, 1600 m	AB123456	–	AB123456
VK0270	*C. nervosus*	Pahang, Tanah Rata, Gn. Jasar, 1600 m	AB123456	AB123456	AB123456
AJ0065	*C. nervosus*	Pahang, Gn. Beremban, 1480 m	AB123456	AB123456	AB123456
AJ0010	*C. nervosus*	Pahang, Gn. Beremban, 1480 m	AB123456	AB123456	AB123456
AJ0080	*C. nervosus*	Pahang, Gn. Beremban, 1480 m	AB123456	AB123456	AB123456
VK0250	*C. nervosus*	Pahang, Tanah Rata, Gn. Jasar, 1600 m	–	–	AB123456
AJ0085	*C. nervosus*	Pahang, Brinchang, Gn. Brinchang, 1800 m	AB123456	AB123456	AB123456
AJ0086	*C. nervosus*	Pahang, Brinchang, Gn. Brinchang, 1800 m	AB123456	AB123456	AB123456
VK0566	*C. nervosus*	Pahang, Tanah Rata, Gn. Jasar, 1600 m	AB123456	–	AB123456
VK0567	*C. nervosus*	Pahang, Tanah Rata, Gn. Jasar, 1600 m	AB123456	–	AB123456
VK0527	*C. pahangensis*	Pahang, Tanah Rata, 1600 m	AB123456	AB123456	AB123456
AJ0095	*C. paraimitator*	Pahang, 12 km, rd. Kg. Raja–Gua Musang, 980 m	KR140300	AB123456	AB123456
AJ0008	*C. paraimitator*	Pahang, Tioman, Kg. Tekek–Juara, 50–300 m	KR140301	AB123456	AB123456
AJ0009	*C. paraimitator*	Pahang, Tioman, Kg. Tekek–Juara, 50–300 m	KR140302	AB123456	AB123456
AJ0026	*C. parallelus*	Pahang, Kg. Kuala Boh, 960 m	KR140303	AB123456	AB123456
AJ0060	*C. parallelus*	Pahang, Gn. Beremban, 1480 m	KR140304	AB123456	AB123456
MM0006	*C. pauper*	Perak, Ringlet, 800 m	AB123456	AB123456	–
AJ0033	*C. pauper*	Pahang, 12 km, rd. Kg. Raja–Gua Musang, 980 m	KR140288	AB123456	AB123456
AJ0043	*C. pauperulus*	Perak, 24 km, rd. Tapah–Ringlet, 350 m	KR140291	AB123456	AB123456
VK0420	*C. pauperulus*	Pahang, Tanah Rata	KR140320	AB123456	AB123456
AJ0027	*C. renatae*	Pahang, 12 km, rd. Kg. Raja–Gua Musang, 980 m	AB123456	AB123456	AB123456
AJ0035	*C. renatae*	Pahang, 12 km, rd. Kg. Raja–Gua Musang, 980 m	AB123456	AB123456	–
LB0198	*C. renatae*	Pahang, 30 km NE of Raub	AB123456	–	AB123456
AJ0018	*C. reverandi*	Pahang, Kg. Kuala Boh, 1040 m	AB123456	AB123456	AB123456
AJ0024	*C. reverandi*	Pahang, Kg. Kuala Boh, 960 m	AB123456	AB123456	AB123456
AJ0029	*C. reverandi*	Pahang, 12 km, rd. Kg. Raja–Gua Musang, 980 m	AB123456	AB123456	AB123456
AJ0030	*C. reverandi*	Pahang, 12 km, rd. Kg. Raja–Gua Musang, 980 m	AB123456	AB123456	AB123456
AJ0031	*C. reverandi*	Pahang, 12 km, rd. Kg. Raja–Gua Musang, 980 m	AB123456	AB123456	AB123456
LB0178	C. *reverandi*	Pahang, 30 km NE of Raub	KC538285	KC538669	KC538476
VK0430	*C. reverandi*	Perak, Ringlet, 40 km SE of Ipoh, 900 m	AB123456	–	AB123456
MM0018	*C. rianganus*	Sumatra Barat, Lake Maninjau	AB123456	AB123456	AB123456
MM0020	*C. rianganus*	Sumatra Barat, Lake Maninjau	AB123456	–	AB123456
MM0022	*C. rianganus*	Sumatra Barat, Lake Maninjau	AB123456	–	AB123456
MM0023	*C. rianganus*	Sumatra Barat, Lake Maninjau	AB123456	–	AB123456
MM0025	*C. rianganus*	Sumatra Barat, Lake Maninjau	AB123456	–	AB123456
LB0173	*C. rianganus*	Pahang, 30 km NE of Raub	–	–	AB123456
LB0296	*C. rianganus*	Sumatra Barat, Gn. Talamau	–	–	AB123456
LB0297	*C. rianganus*	Sumatra Barat, Gn. Talamau	KC538305	KC538690	KC538496
LB0298	*C. rianganus*	Sumatra Barat, Gn. Talamau	–	–	AB123456
LB0299	*C. rianganus*	Sumatra Barat, Gn. Talamau	–	–	AB123456
AJ0011	*C. simillimus*	Pahang, Gn. Beremban, 1480 m	AB123456	AB123456	AB123456
AJ0073	*C. simillimus*	Pahang, Gn. Beremban, 1480 m	AB123456	AB123456	–
AJ0090	*C. simillimus*	Pahang, Gn. Beremban, N slope, 1580 m	KR140289	AB123456	AB123456
VK0259	*C. simillimus*	Pahang, Tanah Rata, Gn. Jasar, 1500–1800 m	AB123456	–	AB123456
VK0247	*C. simillimus*	Pahang, Tanah Rata, Gn. Jasar, 1500–1800 m	AB123456	AB123456	AB123456
VK0283	*C. simillimus*	Pahang, Tanah Rata, Gn. Jasar, 1500–1800 m	AB123456	–	AB123456
VK0309	*C. simillimus*	Pahang, Tanah Rata, 1400–1650 m	AB123456	AB123456	AB123456
VK0574	*C. simillimus*	Pahang, Tanah Rata, 1600 m	AB123456	–	AB123456
VK0620	*C. simillimus*	Pahang, Tanah Rata, Gn. Jasar, 1600 m	AB123456	–	AB123456
MM0005	*C. simillimus*	Perak, Ringlet	AB123456	AB123456	–

LB0088	*C. tanahratensis*	Pahang, Cameron Highlands	KC538268	KC538654	KC538460
AJ0014	*C. tanahratensis*	Perak, 47 km, rd. Ipoh–Kg. Raja, 1030 m	KR140297	AB123456	AB123456
AJ0051	*C. tapahensis*	Perak, 24 km, rd. Tapah–Ringlet, 350 m	KR140306	AB123456	AB123456
AJ0053	*C. tapahensis*	Perak, 24 km, rd. Tapah–Ringlet, 350 m	KR140308	AB123456	AB123456
AJ0055	*C. tapahensis*	Perak, 24 km, rd. Tapah–Ringlet, 350 m	KR140310	AB123456	AB123456
LB0205	*Cautires* sp. A	Jambi, Gn. Tujuh	KC538291	KC538675	KC538482
MM0061	*Cautires* sp. A	Jambi, Gn. Kerinci	AB123456	–	AB123456
MM0015	*Cautires* sp. B	Sumatra Barat, Lake Maninjau	AB123456	–	AB123456
MM0036	*Cautires* sp. B	Jambi, Kerinci Seblat NP	AB123456	AB123456	AB123456
MM0039	*Cautires* sp. C	Jambi, Kerinci Seblat NP	–	AB123456	AB123456
MM0013	*Cautires* sp. D	Sumatra Barat, Lake Maninjau	AB123456	–	AB123456
MM0017	*Cautires* sp. D	Sumatra Barat, Lake Maninjau	AB123456	–	AB123456
MM0027	*Cautires* sp. D	Sumatra Barat, Lake Pasaman, Gn. Talamau	AB123456	–	AB123456
MM0055	*Cautires* sp. E	Jambi, Gn. Kerinci	AB123456	–	AB123456
MM0056	*Cautires* sp. E	Jambi, Gn. Kerinci	AB123456	–	AB123456
AJ0019	*Cautires* sp. F	Pahang, Kg. Kuala Boh, 960 m	AB123456	AB123456	AB123456
AJ0020	*Cautires* sp. F	Pahang, Kg. Kuala Boh, 960 m	AB123456	AB123456	AB123456
AJ0021	*Cautires* sp. F	Pahang, Kg. Kuala Boh, 960 m	AB123456	AB123456	AB123456
AJ0022	*Cautires* sp. F	Pahang, Kg. Kuala Boh, 960 m	AB123456	AB123456	AB123456
AJ0025	*Cautires* sp. F	Pahang, Kg. Kuala Boh, 960 m	AB123456	AB123456	AB123456
VK0546	*Cautires* sp. G	Sumatra Barat, Lake Maninjau, E coast, 800 m	AB123456	AB123456	AB123456
VK0548	*Cautires* sp. G	Sumatra Barat, Lake Maninjau, E coast, 800 m	AB123456	–	AB123456
LB0174	*Cautires* sp. G	Pahang, 30 km NE of Raub	KC538283	–	KC538474
LB0197	*Cautires* sp. G	Pahang, 30 km NE of Raub	–	–	AB123456
LB0061	*Cautires* sp. G	Sumatra Barat, Gn. Merapi, 1050 m	–	–	AB123456
LB0056	*Cautires* sp. H	Jambi, Gn. Tujuh	KC538254	KC538641	KC538446
VK0582	*Cautires* sp. H	Jambi, Kersik Tua, Gn. Kerinci, 1800m	AB123456	AB123456	AB123456
LB0054	*Cautires* sp. I	Jambi, Gn. Tujuh	AB123456	–	AB123456
LB0057	*Cautires* sp. I	Jambi, Gn. Tujuh	–	–	AB123456
LB0052	*Cautires* sp. J	Jambi, Gn. Tujuh	KC538253	KC538640	KC538445
VK0216	*Cautires* sp. K	Sumatra Utara, Brastagi; Gn. Sinabung, 1800 m	AB123456	–	AB123456
VK0218	*Cautires* sp. K	Sumatra Utara, Brastagi; Gn. Sinabung, 1800 m	AB123456	–	AB123456
VK0219	*Cautires* sp. K	Sumatra Utara, Brastagi; Gn. Sinabung, 1800 m	AB123456	AB123456	AB123456
VK0409	*Cautires* sp. K	Sumatra Utara, Brastagi, Gn. Sibayak, 1800 m	AB123456	–	AB123456
VK0444	*Cautires* sp. K	Sumatra Utara, Brastagi, Gn. Sibayak, 1800m	AB123456	–	AB123456
MW0026	*Cautires* sp. K	Sumatra Utara, Brastagi, Gn. Sibayak	AB123456	AB123456	AB123456
VK0529	*Cautires* sp. L	Pahang, Tanah Rata, 1600 m	AB123456	AB123456	AB123456
VK0554	*Cautires* sp. L	Johor, 20 km N of Kotatinggi	AB123456	–	AB123456
VK0215	*Cautires* sp. M	Sumatra Utara, Brastagi; Gn. Sinabung, 1600 m	AB123456	AB123456	AB123456
VK0404	*Cautires* sp. M	Sumatra Utara, Brastagi, Gn. Sibayak, 1800m	AB123456	–	AB123456
VK0407	*Cautires* sp. M	Sumatra Utara, Brastagi, Gn. Sibayak, 1800m	AB123456	–	AB123456
VK0439	*Cautires* sp. M	Sumatra Utara, Brastagi, Gn. Sibayak, 1800m	AB123456	AB123456	AB123456
VK0441	*Cautires* sp. M	Sumatra Utara, Brastagi, Gn. Sibayak, 1800m	AB123456	–	AB123456
VK0446	*Cautires* sp. M	Sumatra Utara, Brastagi, Gn. Sibayak, 1800m	AB123456	–	AB123456
VK0447	*Cautires* sp. M	Sumatra Utara, Brastagi, Gn. Sibayak, 1800m	AB123456	–	AB123456
VK0205	Cautires sp. N	Sumatra Barat, Gn. Merapi, 1600 m	AB123456	–	AB123456
VK0221	*Cautires* sp. N	Sumatra Utara, Brastagi, Gn. Sinabung, 1600 m	AB123456	–	–
VK0405	*Cautires* sp. N	Sumatra Utara, Brastagi, Gn. Sibayak, 1800m	AB123456	–	AB123456
VK0411	*Cautires* sp. N	Sumatra Utara, Brastagi, Gn. Sibayak, 1800m	AB123456	–	AB123456
VK0412	*Cautires* sp. N	Sumatra Utara, Brastagi, Gn. Sibayak, 1800m	AB123456	AB123456	AB123456
VK0440	*Cautires* sp. N	Sumatra Utara, Brastagi, Gn. Sibayak, 1800m	AB123456	–	AB123456
VK0503	*Cautires* sp. N	Jambi, Kersik Tua, Gn. Kerinci, 1800m	AB123456	–	–
VK0501	*Cautires* sp. N	Jambi, Kersik Tua, Gn. Kerinci, 1800m	AB123456	AB123456	AB123456
VK0502	*Cautires* sp. N	Jambi, Kersik Tua, Gn. Kerinci, 1800m	AB123456	–	AB123456
VK0225	*Cautires* sp. N	Sumatra, 7km E of Kayu Aro, Gn. Tujuh, 1750m	AB123456	–	–
VK0182	*Cautires* sp. N	Jambi, Kersik Tua, Gn. Kerinci, 1800m	AB123456	–	AB123456
VK0579	*Cautires* sp. N	Jambi, Kersik Tua, Gn. Kerinci, 1800m	AB123456	–	AB123456
VK0581	*Cautires* sp. N	Jambi, Kersik Tua, Gn. Kerinci, 1800m	AB123456	–	–
LB0047	*Cautires* sp. N	Jambi, Gn. Tujuh	KC538250	KC538637	KC538442
LB0051	*Cautires* sp. N	Jambi, Gn. Tujuh	–	–	AB123456
LB0053	*Cautires* sp. N	Jambi, Gn. Tujuh	–	–	AB123456
LB0055	*Cautires* sp. N	Jambi, Gn. Tujuh	–	–	AB123456
VK0223	*Cautires* sp. O	Sumatra Utara, Brastagi, Gn. Sinabung, 1600 m	AB123456	–	AB123456
VK0402	*Cautires* sp. O	Sumatra Utara, Brastagi, Gn. Sibayak, 1800m	AB123456	–	AB123456
VK0403	*Cautires* sp. O	Sumatra Utara, Brastagi, Gn. Sibayak, 1800m	AB123456	AB123456	AB123456
VK0410	*Cautires* sp. O	Sumatra Utara, Brastagi, Gn. Sibayak, 1800m	AB123456	–	AB123456
VK0442	*Cautires* sp. O	Sumatra Utara, Brastagi, Gn. Sibayak, 1800m	AB123456	–	AB123456
VK0491	*Cautires* sp. O	Sumatra Utara, Brastagi, Gn. Sibayak, 1800m	AB123456	AB123456	AB123456
VK0492	*Cautires* sp. O	Sumatra Utara, Brastagi, Gn. Sibayak, 1800m	AB123456	–	AB123456
VK0547	Cautires sp. O	Sumatra Barat, Lake Maninjau, E coast 800 m	AB123456	–	AB123456
LB0123	*Cautires* sp. P	Java, Trawas, Gn. Penanggungan	KC538276	AB123456	AB123456
VK0220	*Cautires* sp. Q	Sumatra Utara, Brastagi, Gn. Sinabung, 1600 m	AB123456	–	AB123456
VK0438	*Cautires* sp. Q	Sumatra Utara, Brastagi, Gn. Sibayak, 1800m	AB123456	AB123456	AB123456
VK0443	*Cautires* sp. Q	Sumatra Utara, Brastagi, Gn. Sibayak, 1800m	AB123456	–	AB123456
AJ0077	*Cautires* sp. R	Pahang, Gn. Beremban, 1480 m	AB123456	AB123456	AB123456
AJ0036	*Cautires* sp. S	Pahang, 12 km, rd. Kg. Raja–Gua Musang, 980 m	AB123456	AB123456	AB123456
VK0424	*Cautires* sp. S	Pahang, Tanah Rata, 1600 m	AB123456	AB123456	AB123456

VK0448	*Cautires* sp. T	Pahang, Tanah Rata	AB123456	–	AB123456
AJ0012	*Cautires* sp. T	Pahang, Gn. Beremban, 1320 m	AB123456	AB123456	AB123456
AJ0016	*Cautires* sp. T	Pahang, Gn. Jasar, N slope, 1550 m	AB123456	AB123456	AB123456
AJ0063	*Cautires* sp. T	Pahang, Gn. Beremban, 1480 m	AB123456	AB123456	AB123456
VK0583	*Cautires* sp. T	Pahang, Tanah Rata, Gn. Jasar, 1500–1800 m	AB123456	–	AB123456
VK0584	*Cautires* sp. T	Pahang, Tanah Rata, Gn. Jasar, 1500–1800 m	AB123456	–	AB123456
VK0585	*Cautires* sp. T	Pahang, Tanah Rata, Gn. Jasar, 1500–1800 m	AB123456	–	AB123456
VK0586	*Cautires* sp. T	Pahang, Tanah Rata, Gn. Jasar, 1500–1800 m	AB123456	–	AB123456
VK0587	*Cautires* sp. T	Pahang, Tanah Rata, Gn. Jasar, 1500–1800 m	AB123456	–	AB123456
VK0588	*Cautires* sp. T	Pahang, Tanah Rata, Gn. Jasar, 1500–1800 m	AB123456	–	AB123456
VK0589	*Cautires* sp. T	Pahang, Tanah Rata, Gn. Jasar, 1500–1800 m	AB123456	–	AB123456
VK0590	*Cautires* sp. T	Pahang, Tanah Rata, Gn. Jasar, 1500–1800 m	AB123456	–	AB123456
VK0591	*Cautires* sp. T	Pahang, Tanah Rata, Gn. Jasar, 1500–1800 m	AB123456	–	AB123456
VK0592	*Cautires* sp. T	Pahang, Tanah Rata, Gn. Jasar, 1500–1800 m	AB123456	–	AB123456
VK0593	*Cautires* sp. T	Pahang, Tanah Rata, Gn. Jasar, 1500–1800 m	AB123456	–	AB123456
VK0594	*Cautires* sp. T	Pahang, Tanah Rata, Gn. Jasar, 1500–1800 m	AB123456	–	AB123456
VK0595	*Cautires* sp. T	Pahang, Tanah Rata, Gn. Jasar, 1500–1800 m	AB123456	–	AB123456
AJ0074	*Cautires* sp. T	Pahang, Tanah Rata, 1400–1600 m	AB123456	AB123456	–
AJ0075	*Cautires* sp. T	Pahang, Gn. Jasar, E slope, 1500 m	AB123456	AB123456	–
AJ0081	*Cautires* sp. T	Pahang, Brinchang, Gn. Brinchang, 1800 m	AB123456	AB123456	AB123456
VK0522	*Cautires* sp. T	Pahang, Tanah Rata, Gn. Jasar, 1500–1800 m	AB123456	–	AB123456
VK0528	*Cautires* sp. T	Pahang, Tanah Rata, 1600 m	AB123456	–	AB123456
VK0530	*Cautires* sp. T	Pahang, Tanah Rata, 1600 m	AB123456	AB123456	AB123456
VK0531	*Cautires* sp. T	Pahang, Tanah Rata, 1600 m	AB123456	AB123456	AB123456
VK0532	*Cautires* sp. T	Pahang, Tanah Rata, 1600 m	AB123456	–	AB123456
VK0624	*Cautires* sp. T	Pahang, Tanah Rata	AB123456	–	AB123456
VK0626	*Cautires* sp. T	Pahang, Tanah Rata	AB123456	–	AB123456
LB0070	*Cautires* sp. T	Pahang, Tanah Rata	KC538260	KC538647	KC538452
VK0214	*Cautires* sp. U	Jambi, Kersik Tua, Gn. Kerinci, 1800m	AB123456	–	AB123456
VK0229	*Cautires* sp. U	Jambi, 7km E of Kayu Aro Gn. Tujuh,1750 m	AB123456	AB123456	AB123456
VK0256	*Cautires* sp. V	Pahang, Tanah Rata, Gn. Jasar, 1500–1800 m	AB123456	AB123456	AB123456
VK0320	*Cautires* sp. W	Pahang, Tanah Rata, Gn. Jasar, 1500–1800 m	AB123456	AB123456	AB123456
AJ0037	*Cautires* sp. X	Perak, 24 km, rd. Tapah–Ringlet, 350 m	AB123456	AB123456	AB123456
LB0060	*Cautires* sp. Y	Sumatra Barat, Gn. Merapi	KC538255	KC538642	KC538447
VK0577	*Cautires* sp. Z	Jambi, Kersik Tua, Gn. Kerinci, 1800m	AB123456	–	AB123456
VK0578	*Cautires* sp. Z	Jambi, Kersik Tua, Gn. Kerinci, 1800m	AB123456	AB123456	AB123456
VK0580	*Cautires* sp. Z	Jambi, Kersik Tua, Gn. Kerinci, 1800m	AB123456	–	AB123456
LB0049	*Cautires* sp. AA	Jambi, Gn. Tujuh	–	–	AB123456
LB0050	*Cautires* sp. AA	Jambi, Gn. Tujuh	KC538252	KC538639	KC538444
VK0206	*Cautires* sp. AA	Jambi, Kersik Tua, Gn. Kerinci, 1800m	AB123456	AB123456	AB123456
VK0228	*Cautires* sp. AA	Jambi, 7km E of Kayu Aro Gn. Tujuh, 1750 m	AB123456	–	AB123456
VK0408	*Cautires* sp. AB	Sumatra Utara, Brastagi, Gn. Sibayak, 1700 m	AB123456	AB123456	AB123456
VK0413	*Cautires* sp. AB	Sumatra Utara, Brastagi, Gn. Sibayak, 1700 m	AB123456	–	AB123456
VK0226	*Cautires* sp. AC	Jambi, 7km E of Kayu Aro Gn. Tujuh, 1750 m	AB123456	AB123456	AB123456
VK0222	*Cautires* sp. AD	Sumatra Utara, Brastagi, Gn. Sinabung, 1700 m	AB123456	AB123456	AB123456
LB0072	*Cautires* sp. AD	Sumatra Barat, Gn. Talamau	–	–	AB123456
VK0230	*Cautires* sp. AE	Jambi, 7km E of Kayu Aro Gn. Tujuh, 1750 m	AB123456	–	–
VK0231	*Cautires* sp. AE	Jambi, 7km E of Kayu Aro Gn. Tujuh, 1750 m	AB123456	AB123456	AB123456
LB0058	*Cautires* sp. AE	Jambi, Gn. Tujuh	AB123456	–	AB123456
VK0203	*Cautires* sp. AF	Sumatra Barat, Gn. Merapi, 1600 m	AB123456	AB123456	AB123456
VK0204	*Cautires* sp. AF	Sumatra Barat, Gn. Merapi, 1600 m	AB123456	–	AB123456
LB0059	*Cautires* sp. AF	Sumatra Barat, Gn. Merapi	AB123456	–	AB123456
LB0048	*Cautires* sp. AG	Jambi, Gn. Tujuh	KC538251	KC538638	KC538443
VK0212	*Cautires* sp. AG	Jambi, Kersik Tua, Gn. Kerinci, 1800m	AB123456	–	AB123456
VK0211	*Cautires* sp. AH	Jambi, Kersik Tua, Gn. Kerinci, 1800m	AB123456	–	AB123456
VK0213	*Cautires* sp. AH	Jambi, Kersik Tua, Gn. Kerinci, 1800m	AB123456	–	AB123456
VK0207	*Cautires* sp. AH	Jambi, Kersik Tua, Gn. Kerinci, 1800m	AB123456	AB123456	AB123456
VK0208	*Cautires* sp. AH	Jambi, Kersik Tua, Gn. Kerinci, 1800m	AB123456	–	AB123456
VK0209	*Cautires* sp. AH	Jambi, Kersik Tua, Gn. Kerinci, 1800m	AB123456	–	AB123456
VK0210	*Cautires* sp. AH	Jambi, Kersik Tua, Gn. Kerinci, 1800m	AB123456	–	–
VK0224	Cautires sp. AH	Jambi, 7km E of Kayu Aro Gn. Tujuh, 1750 m	AB123456	–	AB123456
VK0227	Cautires sp. AH	Jambi, 7km E of Kayu Aro Gn. Tujuh, 1750 m	AB123456	–	AB123456
VK0497	*Cautires* sp. AH	Jambi, Kersik Tua, Gn. Kerinci, 1800m	AB123456	–	AB123456
VK0498	*Cautires* sp. AH	Jambi, Kersik Tua, Gn. Kerinci, 1800m	AB123456	–	AB123456
VK0499	*Cautires* sp. AH	Jambi, Kersik Tua, Gn. Kerinci, 1800m	AB123456	–	AB123456
VK0500	*Cautires* sp. AH	Jambi, Kersik Tua, Gn. Kerinci, 1800m	AB123456	–	AB123456
MM0060	*Cautires* sp. AH	Jambi, Gn. Kerinci	AB123456	AB123456	–
LB0176	*Cautires* sp. AI	Pahang, 30 km NE of Raub	–	–	AB123456
MM0041	*Cautires* sp. AJ	Jambi, Kerinci Seblat NP	AB123456	AB123456	AB123456
MM0014	*Cautires* sp. AK	Sumatra Barat, Lake Maninjau	AB123456	–	AB123456
MM0019	*Cautires* sp. AK	Sumatra Barat, Lake Maninjau	AB123456	–	AB123456
MM0024	*Cautires* sp. AK	Sumatra Barat, Lake Maninjau	AB123456	–	AB123456
MM0026	*Cautires* sp. AK	Sumatra Barat, Lake Maninjau	AB123456	AB123456	AB123456
LB0295	Cautires sp. AK	Sumatra Barat, Gn. Talamau	KC538304	KC538689	KC538495
MM0040	*Cautires* sp. AL	Jambi, Kerinci Seblat NP	AB123456	AB123456	AB123456
VK0427	*Cautires* sp. AL	Jambi, , 24km NE Tapan Muara Sako, 480 m	AB123456	AB123456	AB123456
AJ0044	*Cautires* sp. AM	Perak, 24 km, rd. Tapah–Ringlet, 350 m	KR140292	AB123456	AB123456

VK0434	*Cautires* sp. AM	Pahang, 30 km NE Raub, Lata Lembik, 300 m	KR140321	AB123456	–
LB0177	Cautires sp. AM	Pahang, 30 km NE of Raub	KC538284	KC538668	KC538475
MM0034	Cautires sp. AM	Pahang, Tanah Rata	AB123456	AB123456	AB123456
MM0058	*Cautires* sp. AN	Jambi, Gn. Kerinci	AB123456	AB123456	AB123456
MM0016	*Cautires* sp. AN	Sumatra Barat, Lake Maninjau	AB123456	–	AB123456
LB0314	*Cautires* sp. AN	Sumatra Barat, Gn. Merapi	KC538306	KC538691	KC538497
AJ0049	*Cautires* sp. AO	Perak, 24 km, rd. Tapah–Ringlet, 350 m	KR140296	AB123456	AB123456
MM0038	*Cautires* sp. AP	Jambi, Kerinci Seblat NP	AB123456	AB123456	AB123456
VK0406	Cautires sp. AQ	Sumatra Utara, Gn. Sibayak, 1800 m	AB123456	AB123456	AB123456
LB0315	*Cautires* sp. AR	Sumatra Barat, Gn. Merapi	KC538307	KC538692	KC538498
LB0316	*Cautires* sp. AR	Sumatra Barat, Gn. Merapi	–	–	AB123456
LB0206	*Cautires* sp. AS	Jambi, Kersik Tua, Gn. Kerinci, 1800 m	KC538292	KC538676	KC538483
MM0059	*Cautires* sp. AT	Jambi, Gn. Kerinci	AB123456	AB123456	AB123456
MM0035	*Cautires* sp. AU	Jambi, Kerinci Seblat NP	AB123456	AB123456	AB123456
MM0037	*Cautires* sp. AU	Jambi, Kerinci Seblat NP	AB123456	AB123456	AB123456
VK0315	*Cautires* sp. AV	Pahang, Tanah Rata, Gn. Jasar, 1500–1800 m	AB123456	–	AB123456
LB0294	*Cautires* sp. AW	Sumatra Barat, Gn. Talamau	KC538303	KC538688	KC538494
MM0042	*Cautires* sp. AX	Sumatra Utara, Brastagi, Gn. Sibayak	AB123456	–	AB123456
MM0043	*Cautires* sp. AX	Sumatra Utara, Brastagi, Gn. Sibayak	AB123456	–	AB123456
MM0044	*Cautires* sp. AX	Sumatra Utara, Brastagi, Gn. Sibayak	AB123456	–	AB123456
MM0045	*Cautires* sp. AX	Sumatra Utara, Brastagi, Gn. Sibayak	AB123456	–	AB123456
MM0046	*Cautires* sp. AX	Sumatra Utara, Brastagi, Gn. Sibayak	AB123456	–	AB123456
MM0047	*Cautires* sp. AX	Sumatra Utara, Brastagi, Gn. Sibayak	AB123456	–	AB123456
MM0048	*Cautires* sp. AX	Sumatra Utara, Brastagi, Gn. Sibayak	AB123456	–	–
MM0049	*Cautires* sp. AX	Sumatra Utara, Brastagi, Gn. Sibayak	AB123456	–	AB123456
MM0050	*Cautires* sp. AX	Sumatra Utara, Brastagi, Gn. Sibayak	AB123456	–	AB123456
MM0051	*Cautires* sp. AX	Sumatra Utara, Brastagi, Gn. Sibayak	AB123456	–	AB123456
MM0052	*Cautires* sp. AX	Sumatra Utara, Brastagi, Gn. Sibayak	AB123456	–	AB123456
MM0053	*Cautires* sp. AX	Sumatra Utara, Brastagi, Gn. Sibayak	AB123456	–	AB123456

Supplementary Table S2. Characteristics of concatenated super-matrices: partition schemes and used models of DNA evolution.

Reduced matrix

Partition	Seq	Site	Unique	Infor	Invar	Const	Model
cox1	80	784	390	347	400	400	GTR+F+I+G4
cox1 tRNA	80	60	22	9	47	47	TIM2+F+I+G4
nad5	83	1017	733	619	326	326	TIM+F+I+G4
cox2	79	260	182	146	94	94	TN+F+I+G4
nad1	71	127	92	60	50	50	TIM2+F+I+G4
nad5 tRNA	83	314	185	105	163	163	TPM3u+F+I+G4
rrnl	71	636	285	205	382	382	GTR+F+I+G4
rrnl tRNA	71	69	23	11	50	50	K3Pu+F+I+G4

All samples

Partition	Seq	Site	Unique	Infor	Invar	Const	Model
cox2	364	260	198	166	81	81	TPM2u+F+I+G4
cox1 tRNA	365	60	24	13	45	45	TIM2+F+I+G4
nad5 tRNA	363	301	207	131	146	146	TPM3u+F+I+G4
cox1	365	786	426	387	372	372	GTR+F+I+G4
nad5	363	1017	806	689	291	291	GTR+F+I+G4
rrnl	194	626	308	236	349	349	GTR+F+I+G4
rrnl tRNA	194	69	27	15	46	46	TPM2+F+I+G4
nad1	192	124	91	64	46	46	GTR+F+I+G4

Abbreviations: Unique: Number of unique site patterns, Infor: Number of parsimony-informative sites, Invar: Number of invariant sites, Const: Number of constant sites (can be subset of invariant sites).

Supplementary Table S3. Uncorrected genetic distances in *Cautires cox1* mitochondrial DNA fragment.

Species *Cautires*	Number of specimens	Minimal intraspecific distance (%)	Maximal intraspecific distance (%)	Relative species	Minimal extraspecific distance (%)	Maximal extraspecific distance (%)
C. alexae	2	0.45	-	*C.* sp. K	2.35	2.44
C. andujari	9	0.99	1.35	*C.* sp. R	7.15	7.69
C. arribasae	7	0.45	0.72	*C.* sp. R	4.07	4.43
C. berembanensis	4	0.27	0.54	*C.* sp. AD	11.59	11.68
C. campestris	4	0.09	0.27	*C. jasarensis*	1.08	1.44
C. communis	56	1.08	1.81	*C.* sp. W	4.98	5.52
C. corporaali	3	0.36	0.54	*C.* sp. AW	2.26	2.53
C. griseus	2	0.09	0.09	*C.* sp. U	12.68	12.68
C. imitator	2	0.09	0.09	*C.* sp. AP/*paraimitator*	3.07	3.17
C. indus	16	0.54	0.72	*C.* sp. AO/AW/*corporaali*	11.86	12.22
C. jasarensis	10	0.36	0.63	*C. campestris*	1.08	1.44
C. katarinae	1	-	-	*C.* sp. U	10.32	-
C. kirstenae	1	-	-	*C.* sp. AD	9.6	-
C. kotatinggensis	4	0.45	0.81	*C.* sp. U	10.77	11.5
C. linardi	3	0.09	0.09	*C.* sp. U	15.39	-
C. maseki	3	0.9	0.9	*C. arribasae*	4.89	5.07
C. nervosus	34	0.9	1.26	*C.* sp. T	11.23	11.59
C. pahangensis	1	-	-	*C.* sp. S	5.43	-
C. paraimitator	3	1.72	1.72	*C.* sp. AP	2.17	2.17
C. parallelus	2	0.09	0.09	*C. paraimitator*	11.41	11.5
C. pauper	2	2.17	2.17	*C.* sp. E/sp. D	2.71	4.61
C. pauperulus	2	0.45	0.45	*C.* sp. AN	0.9	0.99
C. renatae	4	0.54	0.72	*C. campestris*	11.05	11.32
C. reverandi	7	0.36	0.63	*C.* sp. Q	1.72	2.08
C. rianganus	6	0.72	0.81	*C. tapahensis*	0.81	0.9
C. simillimus	10	0.63	0.9	*C.* sp. AE	12.13	12.68
C. sp. A	2	0.18	0.18	*C.* sp. U	13.67	-
C. sp. AA	3	0.45	0.54	*C.* sp. Z	10.05	10.32
C. sp. AB	2	0.54	0.54	*C.* sp. Z	11.59	11.68
C. sp. AC	1	-	-	*C.* sp. AD	3.17	-

C. sp. AD	1	-	-	*C.* sp. AC	3.17	-
C. sp. AE	3	0.09	0.09	*C.* sp. Z	11.5	11.59
C. sp. AF	3	0.27	0.36	*C.* sp. AD	12.4	12.5
C. sp. AG	2	0.18	0.18	*C.* sp. U/sp. AH	13.76	13.86
C. sp. AH	13	0.18	0.36	*C.* sp. AE	12.13	12.4
C. sp. AJ	1	-	-	*C.* sp. AK	1.72	-
C. sp. AK	5	0.18	0.27	*C.* sp. AJ	1.72	1.81
C. sp. AL	2	0.09	0.09	*C.* sp. AM	2.8	2.89
C. sp. AM	4	0.9	1.08	*C.* sp. AL	2.8	3.44
C. sp. AN	3	2.17	2.35	*C. pauperulus*	0.9	1.99
C. sp. AO	1	-	-	*C.* sp. AT	11.5	-
C. sp. AP	1	-	-	*C. paraimitator*	2.17	-
C. sp. AQ	1	-	-	*C.* sp. AR	3.8	-
C. sp. AR	1	-	-	*C.* sp. AT	2.71	-
C. sp. AS	1	-	-	*C.* sp. AT	3.07	-
C. sp. AT	1	-	-	*C.* sp. AR	2.71	-
C. sp. AU	2	0	0	*C.* sp. AR	3.62	-
C. sp. AW	1	-	-	*C. corporaali*	2.26	-
C. sp. AX	12	0.45	0.45	*C. rianganus*	3.71	4.07
C. sp. B	2	2.62	2.62	*C.* sp. U	14.03	14.13
C. sp. D	3	0.72	0.81	*C.* sp. E	2.71	2.8
C. sp. E	2	0.09	0.09	*C. pauper*	2.71	2.8
C. sp. F	5	0.54	0.72	*C.* sp. U	11.14	11.41
C. sp. G	3	0.81	0.81	*C.* sp. U	10.23	10.41
C. sp. H	2	0.18	0.18	*C.* sp. I	1.99	2.17
C. sp. I	1	-	-	*C.* sp. H	1.99	-
C. sp. J	1	-	-	*C.* sp. K	2.35	-
C. sp. K	6	0.36	0.45	*C. alexae*/sp. J	2.35	2.44
C. sp. L	2	0.54	0.54	*C. katarinae*	11.23	-
C. sp. M	7	0.9	0.99	*C. katarinae*	10.5	10.86
C. sp. N	14	1.44	1.72	*C. reverandi*	4.52	6.34
C. sp. O	8	0.54	0.72	*C.* sp. Q/*reverandi*	4.52	4.8
C. sp. R	1	-	-	*C. arribasae*	4.07	-
C. sp. S	2	1.17	1.17	*C. pahangensis*	5.43	5.79
C. sp. T	18	0.09	0.27	*C. pahangensis*	6.34	6.52

C. sp. U	2	0.09	0.09	*C.* sp. G/*katarinae*	10.23	13.04
C. sp. V	1	-	-	*C. communis*	11.23	-
C. sp. W	1	-	-	*C. communis*	4.98	-
C. sp. X	1	-	-	*C. kotatinggensis*	12.13	-
C. sp. Y	1	-	-	*C.* sp. Z	11.41	-
C. sp. Z	3	0.27	0.45	*C.* sp. AA	10.05	10.14
C. tanahratensis	2	0.45	0.45	*C.* sp. AS	3.89	3.98
C. tapahensis	3	0.45	0.54	*C. rianganus*	0.81	1.08

Supplementary Table S4.
Log likelihood and Akaike information criterion (AIC) values for every model compared in the BIOGEOBEARS supermodel using RASP 4.0.

	Malaya x Sumatra		All localities	
	LnL	AICc	LnL	AICc
DEC	-98.58	201.3	-204.7	413.5
**DEC+J**	**-73.1**	**152.5**	**-153.7**	**313.8**
DIVALIKE	-107.6	219.4	-204.5	413.2
DIVALIKE+J	-75.82	158	-157	320.4
BAYAREALIKE	-144.3	292.8	-258.7	521.6
BAYAREALIKE+J	-78.39	163.1	-160.3	327

Supplementary Table S5. The list of sequenced ingroup terminals with description of dorsal colour patters.

Vouch. #	Species	Geographic origin	Colour pattern pronotum/elytra
VK0432	*Cautires alexae*	Perak, 40 km SE of Ipoh, 900 m, Ringlet	testaceous / with testaceous, black apex
VK0433	*C. alexae*	Perak, 40 km SE of Ipoh, 900 m, Ringlet	testaceous / with testaceous, black apex
VK0426	*C. andujari*	Pahang, Tanah Rata, Gn. Jasar, 1600 m	black / black
AJ0082	*C. andujari*	Pahang, Brinchang, Gn. Brinchang, 1800 m	black/black
AJ0083	*C. andujari*	Pahang, Brinchang, Gn. Brinchang, 1800 m	black/black
VK0251	*C. andujari*	Pahang, Tanah Rata, Gn. Jasar, 1600 m	black / black
VK0272	*C. andujari*	Pahang, Tanah Rata, Gn. Jasar, 1500–1800 m	black / black
VK0312	*C. andujari*	Pahang, Tanah Rata, Gn. Jasar, 1500–1800 m	black / black
VK0322	*C. andujari*	Pahang, Tanah Rata, Gn. Jasar, 1500–1800 m	black / black
VK0519	*C. andujari*	Pahang, Tanah Rata, Gn. Jasar, 1500–1800 m	black / black
VK0564	*C. andujari*	Pahang, Tanah Rata, Gn. Jasar, 1600 m	black / black
AJ0058	*C. arribasae*	Pahang, Gn. Beremban	black, costae brown/humeral 2/5 with dark red costae
AJ0059	*C. arribasae*	Pahang, Gn. Beremban	black, costae brown/humeral 2/5 with dark red costae
AJ0062	*C. arribasae*	Pahang, Gn. Beremban	black, costae brown/humeral 2/5 with dark red costae
AJ0064	*C. arribasae*	Pahang, Gn. Beremban	black, costae brown/humeral 2/5 with dark red costae
AJ0066	*C. arribasae*	Pahang, Gn. Beremban	black, costae brown/humeral 2/5 with dark red costae
AJ0078	*C. arribasae*	Pahang, Gn. Beremban	black, costae brown/humeral 2/5 with dark red costae
AJ0079	*C. arribasae*	Pahang, Gn. Beremban	black, costae brown/humeral 2/5 with dark red costae
AJ0092	*C. berembanensis*	Pahang, Gn. Beremban, 1480 m	black/black
VK0254	*C. berembanensis*	Pahang, Tanah Rata, Gn. Jasar, 1500–1800 m	black/black
VK0275	*C. berembanensis*	Pahang, Tanah Rata, Gn. Jasar, 1500–1800 m	black/black
VK0278	*C. berembanensis*	Pahang, Tanah Rata, Gn. Jasar, 1500–1800 m	black/black
AJ0096	*C. campestris*	Pahang, 12 km, rd. Kg. Raja–Gua Musang, 980 m	black / reddish brown humeral costae
AJ0032	*C. campestris*	Pahang, 12 km, rd. Kg. Raja–Gua Musang, 980 m	black / reddish brown humeral costae
AJ0039	*C. campestris*	Perak, 24 km, rd. Tapah–Ringlet, 350 m	dark brown / reddish brown humeral costae
AJ0040	*C. campestris*	Perak, 24 km, rd. Tapah–Ringlet, 350 m	black / reddish brown humeral costae
AJ0041	*C. campestris*	Perak, 24 km, rd. Tapah–Ringlet, 350 m	black / reddish brown humeral costae
AJ0004	*C. communis*	Pahang, Gn. Jasar, N slope, 1550 m	black/black
AJ0006	*C. communis*	Pahang, Gn. Jasar, N slope, 1550 m	black/black
AJ0013	*C. communis*	Pahang, Gn. Beremban, 1480 m	black/black
AJ0061	*C. communis*	Pahang, Gn. Beremban, 1480 m	black/black
AJ0093	*C. communis*	Pahang, Gn. Beremban, 1480 m	black/black
AJ0068	*C. communis*	Pahang, Gn. Beremban, 1480 m	black/black
AJ0084	*C. communis*	Pahang, Brinchang, Gn. Brinchang, 1800 m	black/black
AJ0087	*C. communis*	Pahang, Brinchang, Gn. Brinchang, 1800 m	black/black
AJ0091	*C. communis*	Pahang, Gn. Beremban, 1480 m	black/black
VK0242	*C. communis*	Pahang, Tanah Rata, Gn. Jasar, 1500–1800 m	black/black
VK0244	*C. communis*	Pahang, Tanah Rata, Gn. Jasar, 1500–1800 m	black/black
VK0245	*C. communis*	Pahang, Tanah Rata, Gn. Jasar, 1500–1800 m	black/black
VK0246	*C. communis*	Pahang, Tanah Rata, Gn. Jasar, 1500–1800 m	black/black
VK0255	*C. communis*	Pahang, Tanah Rata, Gn. Jasar, 1500–1800 m	black/black
VK0260	*C. communis*	Pahang, Tanah Rata, Gn. Jasar, 1500–1800 m	black/black
VK0261	*C. communis*	Pahang, Tanah Rata, Gn. Jasar, 1500–1800 m	black/black
VK0262	*C. communis*	Pahang, Tanah Rata, Gn. Jasar, 1500–1800 m	black/black
VK0263	*C. communis*	Pahang, Tanah Rata, Gn. Jasar, 1500–1800 m	black/black
VK0264	*C. communis*	Pahang, Tanah Rata, Gn. Jasar, 1500–1800 m	black/black
VK0265	*C. communis*	Pahang, Tanah Rata, Gn. Jasar, 1500–1800 m	black/black
VK0267	*C. communis*	Pahang, Tanah Rata, Gn. Jasar, 1500–1800 m	black/black
VK0271	*C. communis*	Pahang, Tanah Rata, Gn. Jasar, 1500–1800 m	black/black
VK0274	*C. communis*	Pahang, Tanah Rata, Gn. Jasar, 1500–1800 m	black/black
VK0276	*C. communis*	Pahang, Tanah Rata, Gn. Jasar, 1500–1800 m	black/black
VK0277	*C. communis*	Pahang, Tanah Rata, Gn. Jasar, 1500–1800 m	black/black
VK0279	*C. communis*	Pahang, Tanah Rata, Gn. Jasar, 1500–1800 m	black/black
VK0310	*C. communis*	Pahang, Tanah Rata, 1400–1650 m	black/black
VK0311	*C. communis*	Pahang, Tanah Rata, Gn. Jasar, 1500–1800 m	black/black
VK0313	*C. communis*	Pahang, Tanah Rata, Gn. Jasar, 1500–1800 m	black/black
VK0314	*C. communis*	Pahang, Tanah Rata, Gn. Jasar, 1500–1800 m	black/black
VK0316	*C. communis*	Pahang, Tanah Rata, Gn. Jasar, 1500–1800 m	black/black
VK0317	*C. communis*	Pahang, Tanah Rata, Gn. Jasar, 1500–1800 m	black/black
VK0321	*C. communis*	Pahang, Tanah Rata, Gn. Jasar, 1500–1800 m	black/black
VK0323	*C. communis*	Pahang, Tanah Rata, Gn. Jasar, 1500–1800 m	black/black
VK0324	*C. communis*	Pahang, Tanah Rata, Gn. Jasar, 1500–1800 m	black/black
VK0325	*C. communis*	Pahang, Tanah Rata, Gn. Jasar, 1500–1800 m	black/black
VK0512	*C. communis*	Pahang, Tanah Rata, Gn. Jasar, 1500–1800 m	black/black
VK0513	*C. communis*	Pahang, Tanah Rata, Gn. Jasar, 1500–1800 m	black/black
VK0514	*C. communis*	Pahang, Tanah Rata, Gn. Jasar, 1500–1800 m	black/black
VK0515	*C. communis*	Pahang, Tanah Rata, Gn. Jasar, 1500–1800 m	black/black
VK0520	*C. communis*	Pahang, Tanah Rata, Gn. Jasar, 1500–1800 m	black/black
VK0521	*C. communis.*	Pahang, Tanah Rata, Gn. Jasar, 1500–1800 m	black/black
VK0523	*C. communis*	Pahang, Tanah Rata, Gn. Jasar, 1500–1800 m	black/black
VK0524	*C. communis*	Pahang, Tanah Rata, Gn. Jasar, 1500–1800 m	black/black
VK0421	*C. communis*	Pahang, Tanah Rata, 1600 m	black/black
VK0361	*C. communis*	Pahang, Tanah Rata, 1600 m	black/black
VK0572	*C. communis*	Pahang, Tanah Rata, 1600 m	black/black
VK0526	*C. communis*	Pahang, Tanah Rata, 1600 m	black/black
VK0418	*C. communis*	Pahang, Tanah Rata, 1600 m	black/black

VK0268	*C. communis*	Pahang, Tanah Rata	black/black
VK0450	*C. communis*	Pahang, Tanah Rata	black/black
VK0563	*C. communis*	Pahang, Tanah Rata, Gn. Jasar, 1600 m	black/black
VK0568	*C. communis*	Pahang, Tanah Rata, Gn. Jasar, 1600 m	black/black
VK0619	*C. communis*	Pahang, Tanah Rata, Gn. Jasar, 1600 m	black/black
VK0622	*C. communis*	Pahang, Tanah Rata, Gn. Jasar, 1600 m	black/black
VK0623	*C. communis*	Pahang, Tanah Rata, Gn. Jasar, 1600 m	black/black
MM0054	*C. corporaali*	Sumatra Utara, Brastagi, Gn. Sibayak	orange / humeral 1/3 orange, apex black
MM0057	*C. corporaali*	Sumatra Utara, Brastagi	orange / humeral 1/3 orange, apex black
VK0445	*C. corporaali*	Sumatra Utara, Brastagi, Gn. Sibayak, 1800m	orange / humeral 1/3 orange, apex black
VK0308	*C. griseus*	Pahang, rd. Ipoh–Kg. Raja, 35 km E of Ipoh, 800 m	dark brown / elytra brown in humeral half, dark brown apically
VK0575	*C. griseus*	Pahang, Tanah Rata, 1600 m	testaceous / basal half testaceous
AJ0071	*C. imitator*	Pahang, Gn. Beremban, 1480 m	black / black
AJ0072	*C. imitator*	Pahang, Gn. Beremban, 1480 m	black / black
MM0009	*C. imitator*	Cameron Highlands, 1600 m	black / black
AJ0001	*C. indus*	Perak, 40 km, rd. Tapah–Ringlet, 1030 m	black with reddish costae / humeral 3/5 reddish costae
AJ0002	*C. indus*	Perak, 40 km, rd. Tapah–Ringlet, 1030 m	black / humeral 3/5 reddish costae
AJ0003	*C. indus*	Perak, 40 km, rd. Tapah–Ringlet, 1030 m	black / humeral 3/5 reddish costae
AJ0007	*C. indus*	Pahang, Tioman, Kg. Tekek–Juara, 50–300 m	black with reddish costae / humeral 3/5 reddish costae
AJ0015	*C. indus*	Perak, 47 km, Rd. Ipoh–Kg. Raja, 1030 m	black with reddish costae / humeral 3/5 reddish costae
AJ0045	*C. indus*	Perak, 24 km, rd. Tapah–Ringlet, 350 m	black / humeral 3/5 reddish costae
AJ0046	*C. indus*	Perak, 24 km, rd. Tapah–Ringlet, 350 m	black / humeral 3/5 reddish costae
AJ0047	*C. indus*	Perak, 24 km, rd. Tapah–Ringlet, 350 m	black / humeral 3/5 reddish costae
AJ0052	*C. indus*	Perak, 24 km, rd. Tapah–Ringlet, 350 m	black / humeral 3/5 reddish costae
AJ0050	*C. indus*	Perak, 24 km, rd. Tapah–Ringlet, 350 m	black / humeral 3/5 reddish costae
AJ0054	*C. indus*	Perak, 24 km, rd. Tapah–Ringlet, 350 m	black / humeral 3/5 reddish costae
AJ0056	*C. indus*	Perak, 24 km, rd. Tapah–Ringlet, 350 m	black with reddish costae / humeral 3/5 reddish costae
AJ0057	*C. indus*	Perak, 24 km, rd. Tapah–Ringlet, 350 m	black with reddish costae / humeral 3/5 reddish costae
AJ0038	*C. indus*	Perak, 24 km, rd. Tapah–Ringlet, 350 m	black / humeral 3/5 reddish costae
AJ0094	*C. indus*	Pahang, 12 km, rd. Kg. Raja–Gua Musang, 980 m	black with reddish costae / humeral 3/5 reddish costae
MM0007	*C. indus*	Perak, 40 km SE of Ipoh	black / humeral 3/5 reddish costae
AJ0034	*C. jasarensis*	Pahang, 12 km, rd. Kg. Raja–Gua Musang, 980 m	black/ 3/5 dark red costae
AJ0067	*C. jasarensis*	Pahang, Gn. Beremban, 1480 m	black/black
AJ0070	*C. jasarensis*	Pahang, Gn. Beremban, 1480 m	black dark brown pubescence / humeral quarter dark brown black
VK0252	*C. jasarensis*	Pahang, Tanah Rata, Gn. Jasar, 1600 m	black / black
VK0253	*C. jasarensis*	Pahang, Tanah Rata, Gn. Jasar, 1600 m	black / black
VK0362	*C. jasarensis*	Pahang, Tanah Rata, Gn. Jasar, 1600 m	black / black
VK0569	*C. jasarensis*	Pahang, Tanah Rata, Gn. Jasar, 1600 m	black / black
VK0425	*C. jasarensis*	Pahang, Tanah Rata, Gn. Jasar, 1600 m	black / humeral 1/5 of primary costae reddish
VK0437	*C. jasarensis*	Pahang, rd. Ipoh–Kg. Raja, 51 km E of Ipoh, 1100 m	black / black
VK0565	*C. jasarensis*	Pahang, Tanah Rata, Gn. Jasar, 1600 m	black / black
AJ0028	*C. katarinae*	Pahang, km 12, rd. Kg. Raja–Gua Musang, 980 m	orange testaceous/ orange testaceous apical half brown
VK0419	*C. kirstenae*	Pahang, Tanah Rata, 1600 m	black/black
AJ0042	*C. kotatinggensis*	Perak, 24 km, rd. Tapah–Ringlet, 350 m	black, costae brown/humeral half dark brown
AJ0048	*C. kotatinggensis*	Perak, 24 km, rd. Tapah–Ringlet, 350 m	black/humeral half dark brown
A00048	*C. kotatinggensis*	Johor, 30 km N of Kotatinggi	black/humeral half dark brown
A00050	*C. kotatinggensis*	Johor, 30 km N of Kotatinggi	black/humeral half dark brown
AJ0005	*C. linardi*	Pahang, Gn. Jasar, N slope, 1550 m	black / black
VK0451	*C. linardi*	Pahang, Tanah Rata, Gn. Jasar	black / black
VK0625	*C. linardi*	Pahang, Tanah Rata, Gn. Jasar	black / black
AJ0069	*C. maseki*	Pahang, Gn. Beremban, 1480 m	black / black
VK0280	*C. maseki*	Pahang, Tanah Rata, Gn. Jasar, 1500–1800 m	black / black
VK0628	*C. maseki*	Pahang, Tanah Rata, Gn. Jasar, 1600 m	black / black
VK0576	*C. nervosus*	Pahang, Tanah Rata, Gn. Jasar, 1600 m	black / black
VK0573	*C. nervosus*	Pahang, Tanah Rata, Gn. Jasar, 1600 m	black / black
VK0570	*C. nervosus*	Pahang, Tanah Rata, Gn. Jasar, 1600 m	black / black
VK0571	*C. nervosus*	Pahang, Tanah Rata, Gn. Jasar, 1600 m	black / black
VK0360	*C. nervosus*	Pahang, Tanah Rata, Gn. Jasar, 1600 m	black / black
VK0422	*C. nervosus*	Pahang, Tanah Rata, Gn. Jasar, 1600 m	black / black
VK0533	*C. nervosus*	Pahang, Tanah Rata, Gn. Jasar, 1600 m	black / black
AJ0076	*C. nervosus*	Pahang, Gn. Jasar, E slope, 1500 m	black /black
VK0243	*C. nervosus*	Pahang, Tanah Rata, Gn. Jasar, 1500–1800 m	black / black
VK0257	*C. nervosus*	Pahang, Tanah Rata, Gn. Jasar, 1500–1800 m	black / black
VK0258	*C. nervosus*	Pahang, Tanah Rata, Gn. Jasar, 1500–1800 m	black / black
VK0266	*C. nervosus*	Pahang, Tanah Rata, Gn. Jasar, 1500–1800 m	black / black
VK0516	*C. nervosus*	Pahang, Tanah Rata, Gn. Jasar, 1500–1800 m	black / black
VK0517	*C. nervosus*	Pahang, Tanah Rata, Gn. Jasar, 1500–1800 m	black / black
VK0518	*C. nervosus*	Pahang, Tanah Rata, Gn. Jasar, 1500–1800 m	black / black
VK0525	*C. nervosus*	Pahang, Tanah Rata, Gn. Jasar, 1500–1800 m	black / black
VK0241	*C. nervosus*	Pahang, Tanah Rata, Gn. Jasar, 1500–1800 m	black / black
VK0273	*C. nervosus*	Pahang, Tanah Rata, Gn. Jasar, 1500–1800 m	black / black
VK0281	*C. nervosus*	Pahang, Tanah Rata, Gn. Jasar, 1500–1800 m	black / black
VK0282	*C. nervosus*	Pahang, Tanah Rata, Gn. Jasar, 1500–1800 m	black / black
VK0318	*C. nervosus*	Pahang, Tanah Rata, Gn. Jasar, 1500–1800 m	black / black
VK0319	*C. nervosus*	Pahang, Tanah Rata, Gn. Jasar, 1500–1800 m	black / black
VK0621	*C. nervosus*	Pahang, Tanah Rata, Gn. Jasar, 1600 m	black / black
VK0618	*C. nervosus*	Pahang, Tanah Rata, Gn. Jasar, 1600 m	black / black
VK0453	*C. nervosus*	Pahang, Tanah Rata, Gn. Jasar, 1600 m	black / black
VK0269	*C. nervosus*	Pahang, Tanah Rata, Gn. Jasar, 1600 m	black / black
VK0270	*C. nervosus*	Pahang, Tanah Rata, Gn. Jasar, 1600 m	black / black
AJ0065	*C. nervosus*	Pahang, Gn. Beremban, 1480 m	black / black
AJ0010	*C. nervosus*	Pahang, Gn. Beremban, 1480 m	black / black
AJ0080	*C. nervosus*	Pahang, Gn. Beremban, 1480 m	black/ black

VK0250	*C. nervosus*	Pahang, Tanah Rata, Gn. Jasar, 1600 m	black / black
AJ0085	*C. nervosus*	Pahang, Brinchang, Gn. Brinchang, 1800 m	black/black
AJ0086	*C. nervosus*	Pahang, Brinchang, Gn. Brinchang, 1800 m	black/black
VK0566	*C. nervosus*	Pahang, Tanah Rata, Gn. Jasar, 1600 m	black / black
VK0567	*C. nervosus*	Pahang, Tanah Rata, Gn. Jasar, 1600 m	black / black
VK0527	*C. pahangensis*	Pahang, Tanah Rata, 1600 m	orange/1/6 humeri orange
AJ0095	*C. paraimitator*	Pahang, 12 km, rd. Kg. Raja–Gua Musang, 980 m	black with brown costae / elytral half with reddish brown costae
AJ0008	*C. paraimitator*	Pahang, Tioman, Kg. Tekek–Juara, 50–300 m	black with brown costae / elytral half with reddish brown costae
AJ0009	*C. paraimitator*	Pahang, Tioman, Kg. Tekek–Juara, 50–300 m	black / elytral half with reddish brown costae
AJ0026	*C. parallelus*	Pahang, Kg. Kuala Boh, 960 m	black / black
AJ0060	*C. parallelus*	Pahang, Gn. Beremban, 1480 m	black / black
MM0006	*C. pauper*	Perak, Ringlet, 800 m	testaceous / humeral half testaceous, v-shaped border, brown
AJ0033	*C. pauper*	Pahang, 12 km, rd. Kg. Raja–Gua Musang, 980 m	testaceous / humeral half testaceous, v-shaped border, brown
AJ0043	*C. pauperulus*	Perak, 24 km, rd. Tapah–Ringlet, 350 m	black / 2/3 reddish costae
VK0420	*C. pauperulus*	Pahang, Tanah Rata	black / 1/3 reddish costae
AJ0027	*C. renatae*	Pahang, 12 km, rd. Kg. Raja–Gua Musang, 980 m	orange testaceous/ orange testaceous apical half brown
AJ0035	*C. renatae*	Pahang, 12 km, rd. Kg. Raja–Gua Musang, 980 m	orange testaceous/ orange testaceous apical half brown
000198	*C. renatae*	Pahang, 30 km NE of Raub	testaceous / 1/2 testaceous
LB0198	*C. renatae*	Pahang, 30 km NE of Raub	testaceous / 1/2 testaceous
AJ0018	*C. reverandi*	Pahang, Kg. Kuala Boh, 1040 m	orange testaceous / orange testaceous except elytral apex infuscate
AJ0024	*C. reverandi*	Pahang, Kg. Kuala Boh, 960 m	orange testaceous / orange testaceous except elytral apex infuscate
AJ0029	*C. reverandi*	Pahang, 12 km, rd. Kg. Raja–Gua Musang, 980 m	orange testaceous / orange testaceous except elytral apex infuscate
AJ0030	*C. reverandi*	Pahang, 12 km, rd. Kg. Raja–Gua Musang, 980 m	orange testaceous / orange testaceous except elytral apex infuscate
AJ0031	*C. reverandi*	Pahang, 12 km, rd. Kg. Raja–Gua Musang, 980 m	orange testaceous / orange testaceous except elytral apex infuscate
LB0178	C. *reverandi*	Pahang, 30 km NE of Raub	orange testaceous / orange testaceous except elytral apex infuscate
VK0430	*C. reverandi*	Perak, Ringlet, 40 km SE of Ipoh, 900 m	orange testaceous / orange testaceous except elytral 2/5 infuscate
MM0018	*C. rianganus*	Sumatra Barat, Lake Maninjau	orange / black
MM0020	*C. rianganus*	Sumatra Barat, Lake Maninjau	orange with black patch / black
MM0022	*C. rianganus*	Sumatra Barat, Lake Maninjau	brown / black
MM0023	*C. rianganus*	Sumatra Barat, Lake Maninjau	orange / black
MM0025	*C. rianganus*	Sumatra Barat, Lake Maninjau	brown / black
LB0173	*C. rianganus*	Pahang, 30 km NE of Raub	black / reddish brown 2/3
LB0296	*C. rianganus*	Sumatra Barat, Gn. Talamau	with reddish brown pubescence / black
LB0297	*C. rianganus*	Sumatra Barat, Gn. Talamau	black / black
LB0298	*C. rianganus*	Sumatra Barat, Gn. Talamau	dark red pubescence / black
LB0299	*C. rianganus*	Sumatra Barat, Gn. Talamau	dark red pubescence / black
AJ0011	*C. simillimus*	Pahang, Gn. Beremban, 1480 m	black/black
AJ0073	*C. simillimus*	Pahang, Gn. Beremban, 1480 m	black/black
AJ0090	*C. simillimus*	Pahang, Gn. Beremban, N slope, 1580 m	black/black
VK0259	*C. simillimus*	Pahang, Tanah Rata, Gn. Jasar, 1500–1800 m	black/black
VK0247	*C. simillimus*	Pahang, Tanah Rata, Gn. Jasar, 1500–1800 m	black/black
VK0283	*C. simillimus*	Pahang, Tanah Rata, Gn. Jasar, 1500–1800 m	black/black
VK0309	*C. simillimus*	Pahang, Tanah Rata, 1400–1650 m	black/black
VK0574	*C. simillimus*	Pahang, Tanah Rata, 1600 m	black/black
VK0620	*C. simillimus*	Pahang, Tanah Rata, Gn. Jasar, 1600 m	black/black
MM0005	*C. simillimus*	Perak, Ringlet	black / black
LB0088	*C. tanahratensis*	Pahang, Cameron Highlands	dark testaceous/ 2/3 dark testaceous
AJ0014	*C. tanahratensis*	Perak, 47 km, rd. Ipoh–Kg. Raja, 1030 m	black with dark testaceous margin/ humeral half with dark red costae
AJ0051	*C. tapahensis*	Perak, 24 km, rd. Tapah–Ringlet, 350 m	black/humeral 3/5 dark red costae
AJ0053	*C. tapahensis*	Perak, 24 km, rd. Tapah–Ringlet, 350 m	black/humeral 3/5 dark red costae
AJ0055	*C. tapahensis*	Perak, 24 km, rd. Tapah–Ringlet, 350 m	black/humeral 3/5 dark red costae
LB0205	*Cautires* sp. A	Jambi, Gn. Tujuh	orange / orange quarter
MM0061	*Cautires* sp. A	Jambi, Gn. Kerinci	orange / orange quarter
MM0015	*Cautires* sp. B	Sumatra Barat, Lake Maninjau	black / black
MM0036	*Cautires* sp. B	Jambi, Kerinci Seblat NP	black / black
MM0039	*Cautires* sp. C	Jambi, Kerinci Seblat NP	light brown / quarter light brown
MM0013	*Cautires* sp. D	Sumatra Barat, Lake Maninjau	light brown / quarter light brown
MM0017	*Cautires* sp. D	Sumatra Barat, Lake Maninjau	light brown / quarter light brown
MM0027	*Cautires* sp. D	Sumatra Barat, Lake Pasaman, Gn. Talamau	light brown / quarter light brown
MM0055	*Cautires* sp. E	Jambi, Gn. Kerinci	light brown / 1/10 light brown
MM0056	*Cautires* sp. E	Jambi, Gn. Kerinci	light brown / 1/10 light brown
AJ0019	*Cautires* sp. F	Pahang, Kg. Kuala Boh, 960 m	testaceous / humeral quarter with inconspicuous brown pubescence
AJ0020	*Cautires* sp. F	Pahang, Kg. Kuala Boh, 960 m	dark brown with brown margin / black
AJ0021	*Cautires* sp. F	Pahang, Kg. Kuala Boh, 960 m	brown / black
AJ0022	*Cautires* sp. F	Pahang, Kg. Kuala Boh, 960 m	brown with median black patch / basal 1/3 with inconspicuous brown pubescence
AJ0025	*Cautires* sp. F	Pahang, Kg. Kuala Boh, 960 m	brown with median black patch / black
VK0546	*Cautires* sp. G	Sumatra Barat, Lake Maninjau, E coast, 800 m	testaceous brown / 2/5 testaceous brown
VK0548	*Cautires* sp. G	Sumatra Barat, Lake Maninjau, E coast, 800 m	testaceous brown / 2/5 testaceous brown
LB0174	*Cautires* sp. G	Pahang, 30 km NE of Raub	testaceous / 1/2 testaceous
LB0197	*Cautires* sp. G	Pahang, 30 km NE of Raub	testaceous / 1/2 testaceous
LB0061	*Cautires* sp. G	Sumatra Barat, Gn. Merapi, 1050 m	testaceous / 3/4 testaceous
LB0056	*Cautires* sp. H	Jambi, Gn. Tujuh	orange / orange half of elytra black V-apex
VK0582	*Cautires* sp. H	Jambi, Kersik Tua, Gn. Kerinci, 1800m	orange / orange half of elytra black V-apex
LB0054	*Cautires* sp. I	Jambi, Gn. Tujuh	orange / orange half of elytra black V-apex
LB0057	*Cautires* sp. I	Jambi, Gn. Tujuh	orange / orange half of elytra black V-apex
LB0052	*Cautires* sp. J	Jambi, Gn. Tujuh	orange / orange 1/3 elytra black V-apex
VK0216	*Cautires* sp. K	Sumatra Utara, Brastagi; Gn. Sinabung, 1800 m	orange / 1/3 orange, elytra black V-apex
VK0218	*Cautires* sp. K	Sumatra Utara, Brastagi; Gn. Sinabung, 1800 m	orange / 1/3 orange, elytra black V-apex
VK0219	*Cautires* sp. K	Sumatra Utara, Brastagi; Gn. Sinabung, 1800 m	orange / 1/3 orange, elytra black V-apex
VK0409	*Cautires* sp. K	Sumatra Utara, Brastagi, Gn. Sibayak, 1800 m	orange / 1/3 orange, elytra black V-apex
VK0444	*Cautires* sp. K	Sumatra Utara, Brastagi, Gn. Sibayak, 1800m	orange / 1/3 orange, elytra black V-apex
MW0026	*Cautires* sp. K	Sumatra Utara, Brastagi, Gn. Sibayak	orange with black patch / basal tens of costae orange, elytra black

VK0529	*Cautires* sp. L	Pahang, Tanah Rata, 1600 m	brown / 3/5 reddish brown costae
VK0554	*Cautires* sp. L	Johor, 20 km N of Kotatinggi	brown / 3/5 reddish brown costae
VK0215	*Cautires* sp. M	Sumatra Utara, Brastagi; Gn. Sinabung, 1600 m	orange / 1/3 orange, apex black
VK0404	*Cautires* sp. M	Sumatra Utara, Brastagi, Gn. Sibayak, 1800m	orange / 1/3 orange, apex black
VK0407	*Cautires* sp. M	Sumatra Utara, Brastagi, Gn. Sibayak, 1800m	orange / 1/3 orange, apex black
VK0439	*Cautires* sp. M	Sumatra Utara, Brastagi, Gn. Sibayak, 1800m	orange / 1/3 orange, apex black
VK0441	*Cautires* sp. M	Sumatra Utara, Brastagi, Gn. Sibayak, 1800m	orange / 1/3 orange, apex black
VK0446	*Cautires* sp. M	Sumatra Utara, Brastagi, Gn. Sibayak, 1800m	orange / 1/3 orange, apex black
VK0447	*Cautires* sp. M	Sumatra Utara, Brastagi, Gn. Sibayak, 1800m	orange / 1/3 orange, apex black
VK0205	Cautires sp. N	Sumatra Barat, Gn. Merapi, 1600 m	orange / orange half of elytra black V-apex
VK0221	*Cautires* sp. N	Sumatra Utara, Brastagi, Gn. Sinabung, 1600 m	orange / orange half of elytra black V-apex
VK0405	*Cautires* sp. N	Sumatra Utara, Brastagi, Gn. Sibayak, 1800m	orange / orange half of elytra black apex
VK0411	*Cautires* sp. N	Sumatra Utara, Brastagi, Gn. Sibayak, 1800m	orange / orange half of elytra black V-apex
VK0412	*Cautires* sp. N	Sumatra Utara, Brastagi, Gn. Sibayak, 1800m	orange / orange half of elytra black V-apex
VK0440	*Cautires* sp. N	Sumatra Utara, Brastagi, Gn. Sibayak, 1800m	orange / orange half of elytra black V-apex
VK0503	*Cautires* sp. N	Jambi, Kersik Tua, Gn. Kerinci, 1800m	orange / orange half of elytra black V-apex
VK0501	*Cautires* sp. N	Jambi, Kersik Tua, Gn. Kerinci, 1800m	orange / orange half of elytra black V-apex
VK0502	*Cautires* sp. N	Jambi, Kersik Tua, Gn. Kerinci, 1800m	orange / orange half of elytra black V-apex
VK0225	*Cautires* sp. N	Sumatra, 7km E of Kayu Aro, Gn. Tujuh, 1750m	orange / orange half of elytra black V-apex
VK0182	*Cautires* sp. N	Jambi, Kersik Tua, Gn. Kerinci, 1800m	orange / orange half of elytra black V-apex
VK0579	*Cautires* sp. N	Jambi, Kersik Tua, Gn. Kerinci, 1800m	orange / orange half of elytra black V-apex
VK0581	*Cautires* sp. N	Jambi, Kersik Tua, Gn. Kerinci, 1800m	orange / orange half of elytra black V-apex
LB0047	*Cautires* sp. N	Jambi, Gn. Tujuh	orange / orange half of elytra black V-apex
LB0051	*Cautires* sp. N	Jambi, Gn. Tujuh	orange / orange half of elytra black V-apex
LB0053	*Cautires* sp. N	Jambi, Gn. Tujuh	orange / orange half of elytra black V-apex
LB0055	*Cautires* sp. N	Jambi, Gn. Tujuh	orange / orange half of elytra black V-apex
VK0223	*Cautires* sp. O	Sumatra Utara, Brastagi, Gn. Sinabung, 1600 m	orange / 1/3 orange elytra, black apex
VK0402	*Cautires* sp. O	Sumatra Utara, Brastagi, Gn. Sibayak, 1800m	orange / 1/3 orange elytra, black apex
VK0403	*Cautires* sp. O	Sumatra Utara, Brastagi, Gn. Sibayak, 1800m	orange / 1/3 orange elytra, black apex
VK0410	*Cautires* sp. O	Sumatra Utara, Brastagi, Gn. Sibayak, 1800m	orange / 1/3 orange elytra, black apex
VK0442	*Cautires* sp. O	Sumatra Utara, Brastagi, Gn. Sibayak, 1800m	orange / 1/3 orange elytra, black apex
VK0491	*Cautires* sp. O	Sumatra Utara, Brastagi, Gn. Sibayak, 1800m	orange / 1/3 orange elytra, black apex
VK0492	*Cautires* sp. O	Sumatra Utara, Brastagi, Gn. Sibayak, 1800m	orange / 1/3 orange elytra, black apex
VK0547	Cautires sp. O	Sumatra Barat, Lake Maninjau, E coast 800 m	orange / 1/3 orange elytra, black apex
LB0123	*Cautires* sp. P	Java, Trawas, Gn. Penanggungan	yellow / yellow
VK0220	*Cautires* sp. Q	Sumatra Utara, Brastagi, Gn. Sinabung, 1600 m	orange / 1/3 orange elytra, black apex
VK0438	*Cautires* sp. Q	Sumatra Utara, Brastagi, Gn. Sibayak, 1800m	orange / 1/3 orange elytra, black apex
VK0443	*Cautires* sp. Q	Sumatra Utara, Brastagi, Gn. Sibayak, 1800m	orange / 1/3 orange elytra, black apex
AJ0077	*Cautires* sp. R	Pahang, Gn. Beremban, 1480 m	black / humeral quarter costae with reddish pubescence
AJ0036	*Cautires* sp. S	Pahang, 12 km, rd. Kg. Raja–Gua Musang, 980 m	red / humeral third with dark red pubescence
VK0424	*Cautires* sp. S	Pahang, Tanah Rata, 1600 m	orange-red / 1/5 orange, apex black
VK0448	*Cautires* sp. T	Pahang, Tanah Rata	dark red / 1/6 with reddish costae
AJ0012	*Cautires* sp. T	Pahang, Gn. Beremban, 1320 m	black with reddish pubescence / 1/10 dark reddish costae
AJ0016	*Cautires* sp. T	Pahang, Gn. Jasar, N slope, 1550 m	orange / orange humeral quarter
AJ0063	*Cautires* sp. T	Pahang, Gn. Beremban, 1480 m	black with reddish pubescence / 1/10 dark reddish costae
VK0583	*Cautires* sp. T	Pahang, Tanah Rata, Gn. Jasar, 1500–1800 m	dark red / 1/6 with reddish costae
VK0584	*Cautires* sp. T	Pahang, Tanah Rata, Gn. Jasar, 1500–1800 m	light orange / 1/6 with reddish costae
VK0585	*Cautires* sp. T	Pahang, Tanah Rata, Gn. Jasar, 1500–1800 m	red / 1/6 with reddish costae
VK0586	*Cautires* sp. T	Pahang, Tanah Rata, Gn. Jasar, 1500–1800 m	red / 1/6 with reddish costae
VK0587	*Cautires* sp. T	Pahang, Tanah Rata, Gn. Jasar, 1500–1800 m	dark red / 1/6 with reddish costae
VK0588	*Cautires* sp. T	Pahang, Tanah Rata, Gn. Jasar, 1500–1800 m	red / 1/6 with reddish costae
VK0589	*Cautires* sp. T	Pahang, Tanah Rata, Gn. Jasar, 1500–1800 m	red / 1/6 with reddish costae
VK0590	*Cautires* sp. T	Pahang, Tanah Rata, Gn. Jasar, 1500–1800 m	red / 1/6 with reddish costae
VK0591	*Cautires* sp. T	Pahang, Tanah Rata, Gn. Jasar, 1500–1800 m	red / 1/6 with reddish costae
VK0592	*Cautires* sp. T	Pahang, Tanah Rata, Gn. Jasar, 1500–1800 m	red / 1/6 with reddish costae
VK0593	*Cautires* sp. T	Pahang, Tanah Rata, Gn. Jasar, 1500–1800 m	red / 1/8 with reddish costae
VK0594	*Cautires* sp. T	Pahang, Tanah Rata, Gn. Jasar, 1500–1800 m	red / 1/8 with reddish costae
VK0595	*Cautires* sp. T	Pahang, Tanah Rata, Gn. Jasar, 1500–1800 m	red / 1/8 with reddish costae
AJ0074	*Cautires* sp. T	Pahang, Tanah Rata, 1400–1600 m	orange / 1/10 orange costae black
AJ0075	*Cautires* sp. T	Pahang, Gn. Jasar, E slope, 1500 m	orange / 1/10 orange humeri black
AJ0081	*Cautires* sp. T	Pahang, Brinchang, Gn. Brinchang, 1800 m	orange brown / orange humeri
VK0522	*Cautires* sp. T	Pahang, Tanah Rata, Gn. Jasar, 1500–1800 m	red / 1/6 with reddish costae
VK0528	*Cautires* sp. T	Pahang, Tanah Rata, 1600 m	red / 1/6 with reddish costae
VK0530	*Cautires* sp. T	Pahang, Tanah Rata, 1600 m	red / 1/6 with reddish costae
VK0531	*Cautires* sp. T	Pahang, Tanah Rata, 1600 m	red / 1/6 with reddish costae
VK0532	*Cautires* sp. T	Pahang, Tanah Rata, 1600 m	red / 1/6 with reddish costae
VK0624	*Cautires* sp. T	Pahang, Tanah Rata	red / 1/6 with reddish costae
VK0626	*Cautires* sp. T	Pahang, Tanah Rata	red / 1/6 with reddish costae
LB0070	*Cautires* sp. T	Pahang, Tanah Rata	dark brown/ 1/4 brown
VK0214	*Cautires* sp. U	Jambi, Kersik Tua, Gn. Kerinci, 1800m	yellow / yellow
VK0229	*Cautires* sp. U	Jambi, 7km E of Kayu Aro Gn. Tujuh,1750 m	yellow / yellow
VK0256	*Cautires* sp. V	Pahang, Tanah Rata, Gn. Jasar, 1500–1800 m	black / black
VK0320	*Cautires* sp. W	Pahang, Tanah Rata, Gn. Jasar, 1500–1800 m	black / black
AJ0037	*Cautires* sp. X	Perak, 24 km, rd. Tapah–Ringlet, 350 m	orange testaceous / orange testaceous, apical quarter infuscate
LB0060	*Cautires* sp. Y	Sumatra Barat, Gn. Merapi	black with brown pubescence frontally / black with red humeral third of elytral suture
VK0577	*Cautires* sp. Z	Jambi, Kersik Tua, Gn. Kerinci, 1800m	orange / orange half, black V-apex
VK0578	*Cautires* sp. Z	Jambi, Kersik Tua, Gn. Kerinci, 1800m	orange / orange half, black V-apex
VK0580	*Cautires* sp. Z	Jambi, Kersik Tua, Gn. Kerinci, 1800m	orange / orange half, black V-apex
LB0049	*Cautires* sp. AA	Jambi, Gn. Tujuh	yellow / orange-red
LB0050	*Cautires* sp. AA	Jambi, Gn. Tujuh	yellow / orange-red
VK0206	*Cautires* sp. AA	Jambi, Kersik Tua, Gn. Kerinci, 1800m	yellow / orange-red
VK0228	*Cautires* sp. AA	Jambi, 7km E of Kayu Aro Gn. Tujuh, 1750 m	yellow / orange-red

VK0408	*Cautires* sp. AB	Sumatra Utara, Brastagi, Gn. Sibayak, 1700 m	black / orange red elytra
VK0413	*Cautires* sp. AB	Sumatra Utara, Brastagi, Gn. Sibayak, 1700 m	black / orange red elytra
VK0226	*Cautires* sp. AC	Jambi, 7km E of Kayu Aro Gn. Tujuh, 1750 m	orange / orange 1/2 elytra, black V-apex
VK0222	*Cautires* sp. AD	Sumatra Utara, Brastagi, Gn. Sinabung, 1700 m	orange / orange 1/3 elytra, black V-apex
LB0072	*Cautires* sp. AD	Sumatra Barat, Gn. Talamau	orange / orange 1/3 elytra, black V-apex
VK0230	*Cautires* sp. AE	Jambi, 7km E of Kayu Aro Gn. Tujuh, 1750 m	black / black humeral half of elytra, red of epical half of elytra
VK0231	*Cautires* sp. AE	Jambi, 7km E of Kayu Aro Gn. Tujuh, 1750 m	black / black humeral half of elytra, red of apical half of elytra
LB0058	*Cautires* sp. AE	Jambi, Gn. Tujuh	black / black humeral half of elytra, red of epical half of elytra
VK0203	*Cautires* sp. AF	Sumatra Barat, Gn. Merapi, 1600 m	orange / half orange, apical half black
VK0204	*Cautires* sp. AF	Sumatra Barat, Gn. Merapi, 1600 m	orange / half orange, apical half black
LB0059	*Cautires* sp. AF	Sumatra Barat, Gn. Merapi	orange / orange 1/2
LB0048	*Cautires* sp. AG	Jambi, Gn. Tujuh	orange / orange half of elytra V-apex black
VK0212	*Cautires* sp. AG	Jambi, Kersik Tua, Gn. Kerinci, 1800m	orange / orange half of elytra V-apex black
VK0211	*Cautires* sp. AH	Jambi, Kersik Tua, Gn. Kerinci, 1800m	orange / orange
VK0213	*Cautires* sp. AH	Jambi, Kersik Tua, Gn. Kerinci, 1800m	orange / orange
VK0207	*Cautires* sp. AH	Jambi, Kersik Tua, Gn. Kerinci, 1800m	orange / orange
VK0208	*Cautires* sp. AH	Jambi, Kersik Tua, Gn. Kerinci, 1800m	orange / orange
VK0209	*Cautires* sp. AH	Jambi, Kersik Tua, Gn. Kerinci, 1800m	orange / orange
VK0210	*Cautires* sp. AH	Jambi, Kersik Tua, Gn. Kerinci, 1800m	orange / orange
VK0224	Cautires sp. AH	Jambi, 7km E of Kayu Aro Gn. Tujuh, 1750 m	orange / orange
VK0227	Cautires sp. AH	Jambi, 7km E of Kayu Aro Gn. Tujuh, 1750 m	orange / orange
VK0497	*Cautires* sp. AH	Jambi, Kersik Tua, Gn. Kerinci, 1800m	orange / orange
VK0498	*Cautires* sp. AH	Jambi, Kersik Tua, Gn. Kerinci, 1800m	orange / orange
VK0499	*Cautires* sp. AH	Jambi, Kersik Tua, Gn. Kerinci, 1800m	orange / orange
VK0500	*Cautires* sp. AH	Jambi, Kersik Tua, Gn. Kerinci, 1800m	orange / orange
MM0060	*Cautires* sp. AH	Jambi, Gn. Kerinci	orange / 3/4 orange, apex black
LB0176	*Cautires* sp. AI	Pahang, 30 km NE of Raub	black / 5/6 testaceous
MM0041	*Cautires* sp. AJ	Jambi, Kerinci Seblat NP	black / black
MM0014	*Cautires* sp. AK	Sumatra Barat, Lake Maninjau	orange with black patch / black
MM0019	*Cautires* sp. AK	Sumatra Barat, Lake Maninjau	orange with black patch / black
MM0024	*Cautires* sp. AK	Sumatra Barat, Lake Maninjau	orange with black patch / black
MM0026	*Cautires* sp. AK	Sumatra Barat, Lake Maninjau	orange with black patch / costae humeral 1/10 orange, black
LB0295	Cautires sp. AK	Sumatra Barat, Gn. Talamau	orange with black patch / elytral costa 1 orange in humeral quarter, rest elytra black
MM0040	*Cautires* sp. AL	Jambi, Kerinci Seblat NP	black / black
VK0427	*Cautires* sp. AL	Jambi, , 24km NE Tapan Muara Sako, 480 m	black / black
AJ0044	*Cautires* sp. AM	Perak, 24 km, rd. Tapah–Ringlet, 350 m	black / 2/3 orange costae
VK0434	*Cautires* sp. AM	Pahang, 30 km NE Raub, Lata Lembik, 300 m	brown / 4/5 brownish red costae
LB0177	Cautires sp. AM	Pahang, 30 km NE of Raub	dark brown / 3/4 testaceous
MM0034	Cautires sp. AM	Pahang, Tanah Rata	dark brown/ 1/2 testaceous
MM0058	*Cautires* sp. AN	Jambi, Gn. Kerinci	orange / 1/3 orange
MM0016	*Cautires* sp. AN	Sumatra Barat, Lake Maninjau	orange / 1/6 orange
LB0314	*Cautires* sp. AN	Sumatra Barat, Gn. Merapi	orange testaceous with black patch / 1/3 orange testaceous, rest elytra black
AJ0049	*Cautires* sp. AO	Perak, 24 km, rd. Tapah–Ringlet, 350 m	black / 2/3 orange costae
MM0038	*Cautires* sp. AP	Jambi, Kerinci Seblat NP	black / black
VK0406	Cautires sp. AQ	Sumatra Utara, Gn. Sibayak, 1800 m	orange / orange 1/3 elytra apex black
LB0315	*Cautires* sp. AR	Sumatra Barat, Gn. Merapi	orange / orange 1/3
LB0316	*Cautires* sp. AR	Sumatra Barat, Gn. Merapi	orange / orange 1/3
LB0206	*Cautires* sp. AS	Jambi, Kersik Tua, Gn. Kerinci, 1800 m	orange / black
MM0059	*Cautires* sp. AT	Jambi, Gn. Kerinci	orange / orange 1/6
MM0035	*Cautires* sp. AU	Jambi, Kerinci Seblat NP	brown with black patch / brown 1/3 costae
MM0037	*Cautires* sp. AU	Jambi, Kerinci Seblat NP	brown with black patch / brown 1/3 costae
VK0315	*Cautires* sp. AV	Pahang, Tanah Rata, Gn. Jasar, 1500–1800 m	black / black
LB0294	*Cautires* sp. AW	Sumatra Barat, Gn. Talamau	orange / orange 1/3
MM0042	*Cautires* sp. AX	Sumatra Utara, Brastagi, Gn. Sibayak	black / black
MM0043	*Cautires* sp. AX	Sumatra Utara, Brastagi, Gn. Sibayak	black / black
MM0044	*Cautires* sp. AX	Sumatra Utara, Brastagi, Gn. Sibayak	orange / black
MM0045	*Cautires* sp. AX	Sumatra Utara, Brastagi, Gn. Sibayak	black / black
MM0046	*Cautires* sp. AX	Sumatra Utara, Brastagi, Gn. Sibayak	black / black
MM0047	*Cautires* sp. AX	Sumatra Utara, Brastagi, Gn. Sibayak	black / black
MM0048	*Cautires* sp. AX	Sumatra Utara, Brastagi, Gn. Sibayak	black / black
MM0049	*Cautires* sp. AX	Sumatra Utara, Brastagi, Gn. Sibayak	black / black
MM0050	*Cautires* sp. AX	Sumatra Utara, Brastagi, Gn. Sibayak	black / black
MM0051	*Cautires* sp. AX	Sumatra Utara, Brastagi, Gn. Sibayak	orange / black
MM0052	*Cautires* sp. AX	Sumatra Utara, Brastagi, Gn. Sibayak	black / black
MM0053	*Cautires* sp. AX	Sumatra Utara, Brastagi, Gn. Sibayak	black / black

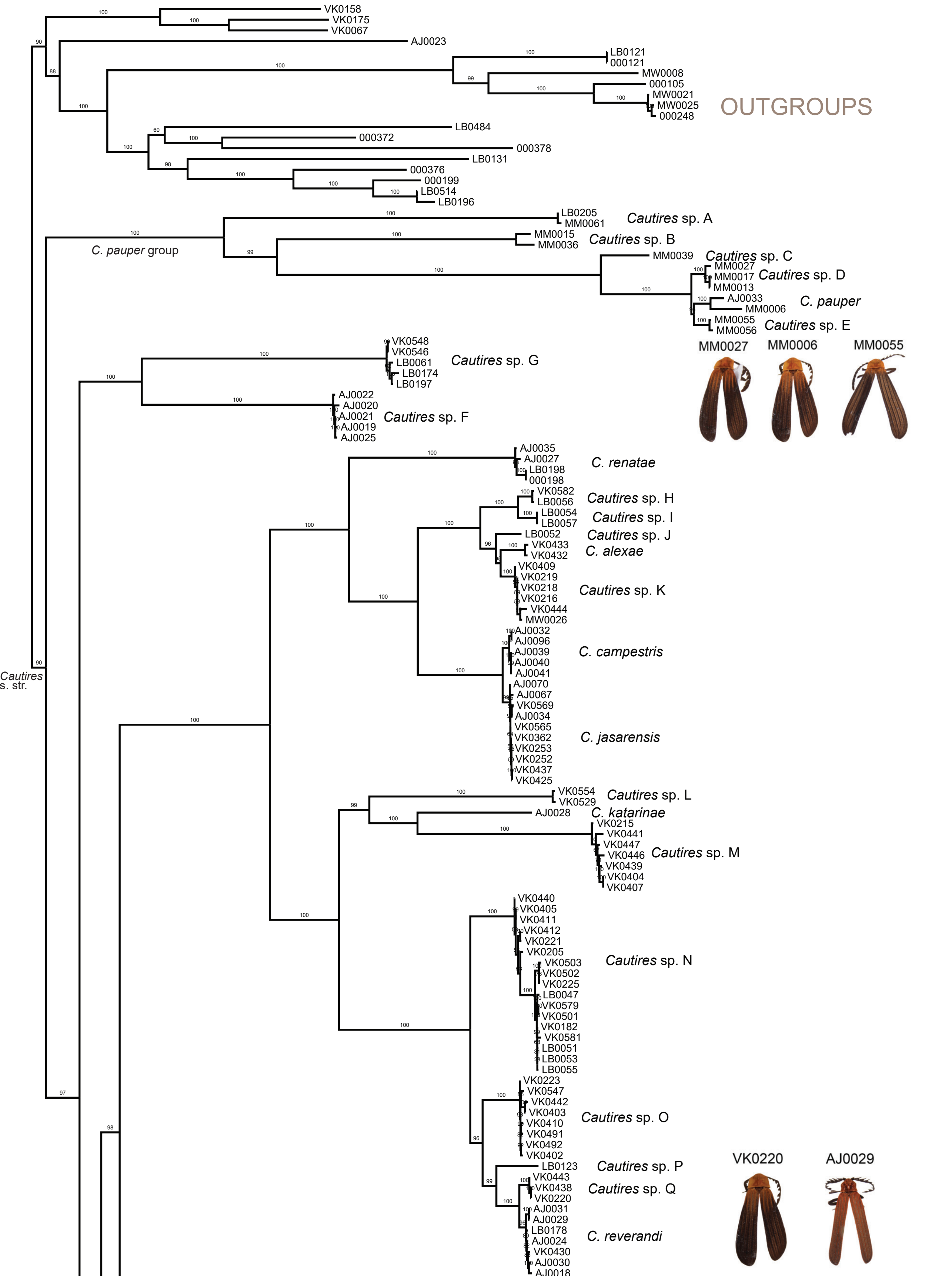
VK0158
VK0175
VK0067
AJ0023
LB0121
000121
MW0008
000105
MW0021
MW0025
000248
OUTGROUPS
LB0484
000372
000378
LB0131
000376
000199
LB0514
LB0196
LB0205
MM0061
Cautires sp. A
MM0015
MM0036
Cautires sp. B
C. pauper group
MM0039
Cautires sp. C
MM0027
MM0017
MM0013
Cautires sp. D
AJ0033
MM0006
C. pauper
MM0055
MM0056
Cautires sp. E
MM0027
MM0006
MM0055
VK0548
VK0546
LB0061
LB0174
LB0197
Cautires sp. G
AJ0022
AJ0020
AJ0021
AJ0019
AJ0025
Cautires sp. F
AJ0035
AJ0027
LB0198
000198
C. renatae
VK0582
LB0056
Cautires sp. H
LB0054
LB0057
Cautires sp. I
LB0052
Cautires sp. J
VK0433
VK0432
C. alexae
VK0409
VK0219
VK0218
VK0216
VK0444
MW0026
Cautires sp. K
AJ0032
AJ0096
AJ0039
AJ0040
AJ0041
C. campestris
AJ0070
AJ0067
VK0569
AJ0034
VK0565
VK0362
VK0253
VK0252
VK0437
VK0425
C. jasarensis
Cautires
s. str.
VK0554
VK0529
Cautires sp. L
AJ0028
C. katarinae
VK0215
VK0441
VK0447
VK0446
VK0439
VK0404
VK0407
Cautires sp. M
VK0440
VK0405
VK0411
VK0412
VK0221
VK0205
VK0503
VK0502
VK0225
LB0047
VK0579
VK0501
VK0182
VK0581
LB0051
LB0053
LB0055
Cautires sp. N
VK0223
VK0547
VK0442
VK0403
VK0410
VK0491
VK0492
VK0402
Cautires sp. O
LB0123
Cautires sp. P
VK0443
VK0438
VK0220
Cautires sp. Q
AJ0031
AJ0029
LB0178
AJ0024
VK0430
AJ0030
AJ0018
C. reverandi
VK0220
AJ0029

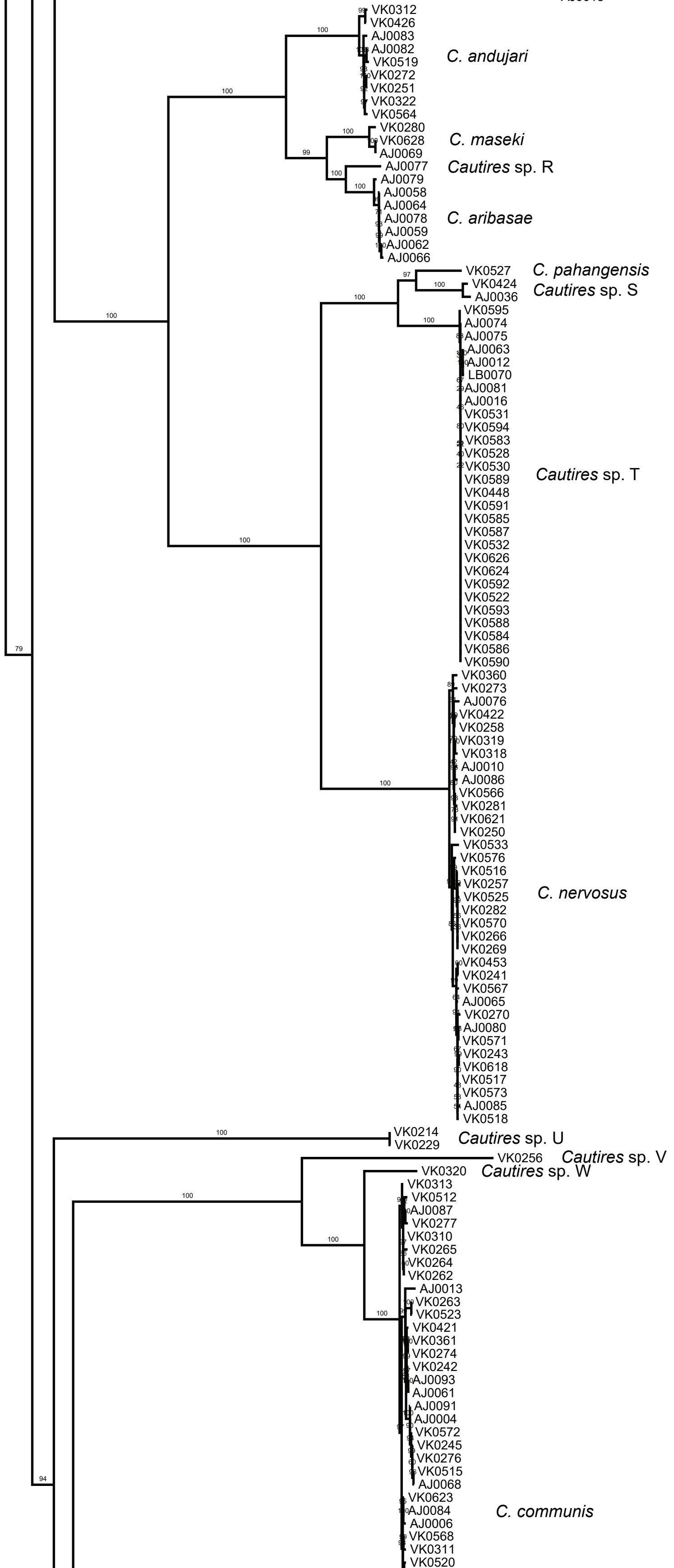
VK0312
VK0426
AJ0083
AJ0082
VK0519
VK0272
VK0251
VK0322
VK0564
C. andujari
VK0280
VK0628
AJ0069
C. maseki
AJ0077
Cautires sp. R
AJ0079
AJ0058
AJ0064
AJ0078
AJ0059
AJ0062
AJ0066
C. aribasae
VK0527
C. pahangensis
VK0424
AJ0036
Cautires sp. S
VK0595
AJ0074
AJ0075
AJ0063
AJ0012
LB0070
AJ0081
AJ0016
VK0531
VK0594
VK0583
VK0528
VK0530
VK0589
VK0448
VK0591
VK0585
VK0587
VK0532
VK0626
VK0624
VK0592
VK0522
VK0593
VK0588
VK0584
VK0586
VK0590
Cautires sp. T
VK0360
VK0273
AJ0076
VK0422
VK0258
VK0319
VK0318
AJ0010
AJ0086
VK0566
VK0281
VK0621
VK0250
VK0533
VK0576
VK0516
VK0257
VK0525
VK0282
VK0570
VK0266
VK0269
VK0453
VK0241
VK0567
AJ0065
VK0270
AJ0080
VK0571
VK0243
VK0618
VK0517
VK0573
AJ0085
VK0518
C. nervosus
VK0214
VK0229
Cautires sp. U
VK0256
Cautires sp. V
VK0320
Cautires sp. W
VK0313
VK0512
AJ0087
VK0277
VK0310
VK0265
VK0264
VK0262
AJ0013
VK0263
VK0523
VK0421
VK0361
VK0274
VK0242
AJ0093
AJ0061
AJ0091
AJ0004
VK0572
VK0245
VK0276
VK0515
AJ0068
VK0623
AJ0084
AJ0006
VK0568
VK0311
VK0520
C. communis
100
100
100
99
100
100
97
100
100
100
100
100
100
79
100
100
100
100
94

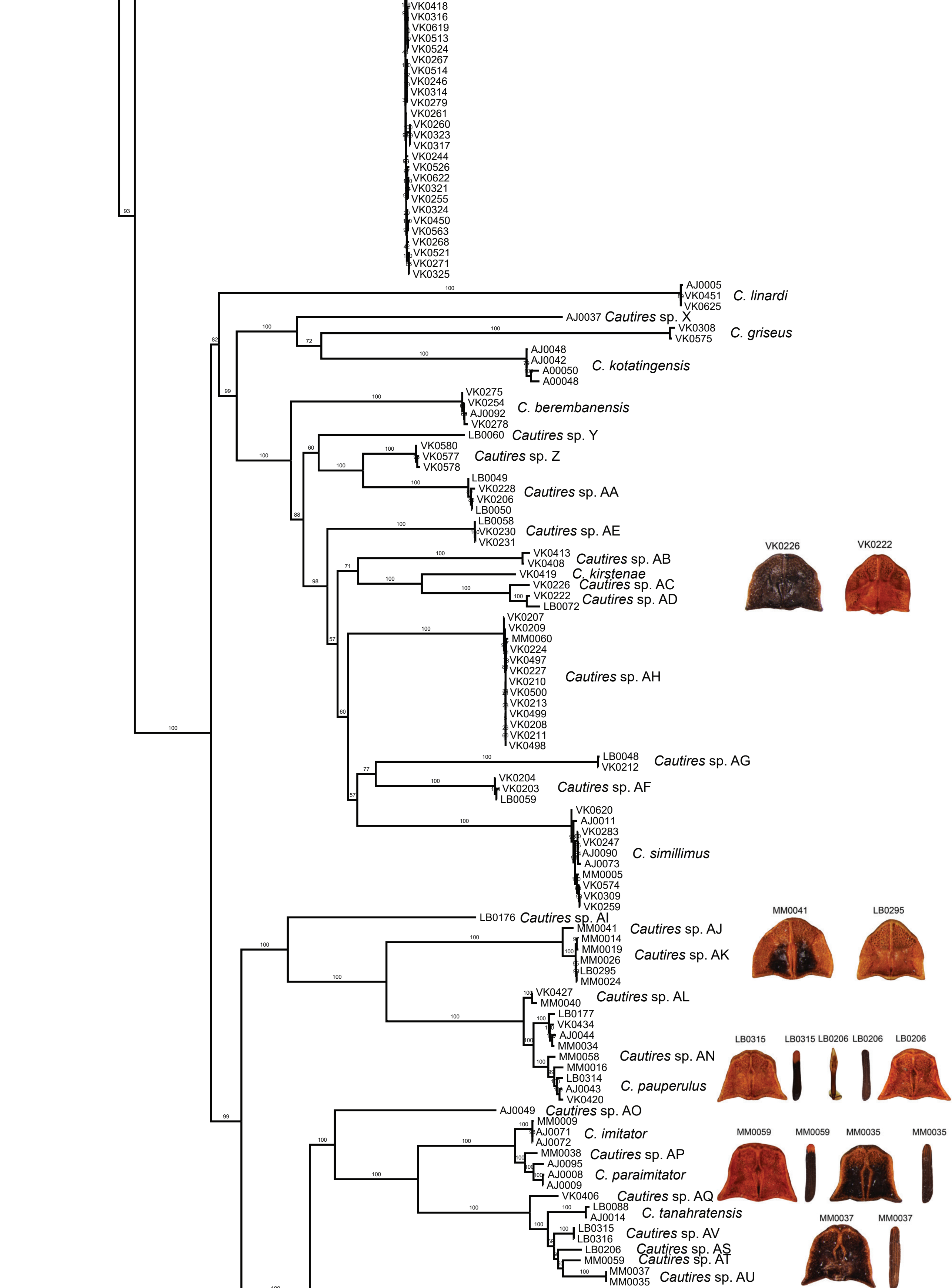

VK0418
VK0316
VK0619
VK0513
VK0524
VK0267
VK0514
VK0246
VK0314
VK0279
VK0261
VK0260
VK0323
VK0317
VK0244
VK0526
VK0622
VK0321
VK0255
VK0324
VK0450
VK0563
VK0268
VK0521
VK0271
VK0325
AJ0005
VK0451
VK0625
C. linardi
AJ0037 Cautires sp. X
VK0308
VK0575
C. griseus
AJ0048
AJ0042
A00050
A00048
C. kotatingensis
VK0275
VK0254
AJ0092
VK0278
C. berembanensis
LB0060 Cautires sp. Y
VK0580
VK0577
VK0578
Cautires sp. Z
LB0049
VK0228
VK0206
LB0050
Cautires sp. AA
LB0058
VK0230
VK0231
Cautires sp. AE
VK0413
VK0408
Cautires sp. AB
VK0419
C. kirstenae
VK0226
Cautires sp. AC
VK0222
LB0072
Cautires sp. AD
VK0207
VK0209
MM0060
VK0224
VK0497
VK0227
VK0210
VK0500
VK0213
VK0499
VK0208
VK0211
VK0498
Cautires sp. AH
LB0048
VK0212
Cautires sp. AG
VK0204
VK0203
LB0059
Cautires sp. AF
VK0620
AJ0011
VK0283
VK0247
AJ0090
AJ0073
MM0005
VK0574
VK0309
VK0259
C. simillimus
LB0176 Cautires sp. AI
MM0041
Cautires sp. AJ
MM0014
MM0019
MM0026
LB0295
MM0024
Cautires sp. AK
VK0427
MM0040
Cautires sp. AL
LB0177
VK0434
AJ0044
MM0034
MM0058
Cautires sp. AN
MM0016
LB0314
AJ0043
VK0420
C. pauperulus
AJ0049 Cautires sp. AO
MM0009
AJ0071
AJ0072
C. imitator
MM0038
Cautires sp. AP
AJ0095
AJ0008
AJ0009
C. paraimitator
VK0406
Cautires sp. AQ
LB0088
AJ0014
C. tanahratensis
LB0315
LB0316
Cautires sp. AV
LB0206
Cautires sp. AS
MM0059
Cautires sp. AT
MM0037
MM0035
Cautires sp. AU
VK0226
VK0222
MM0041
LB0295
LB0315
LB0315
LB0206
LB0206
LB0206
MM0059
MM0059
MM0035
MM0035
MM0037
MM0037

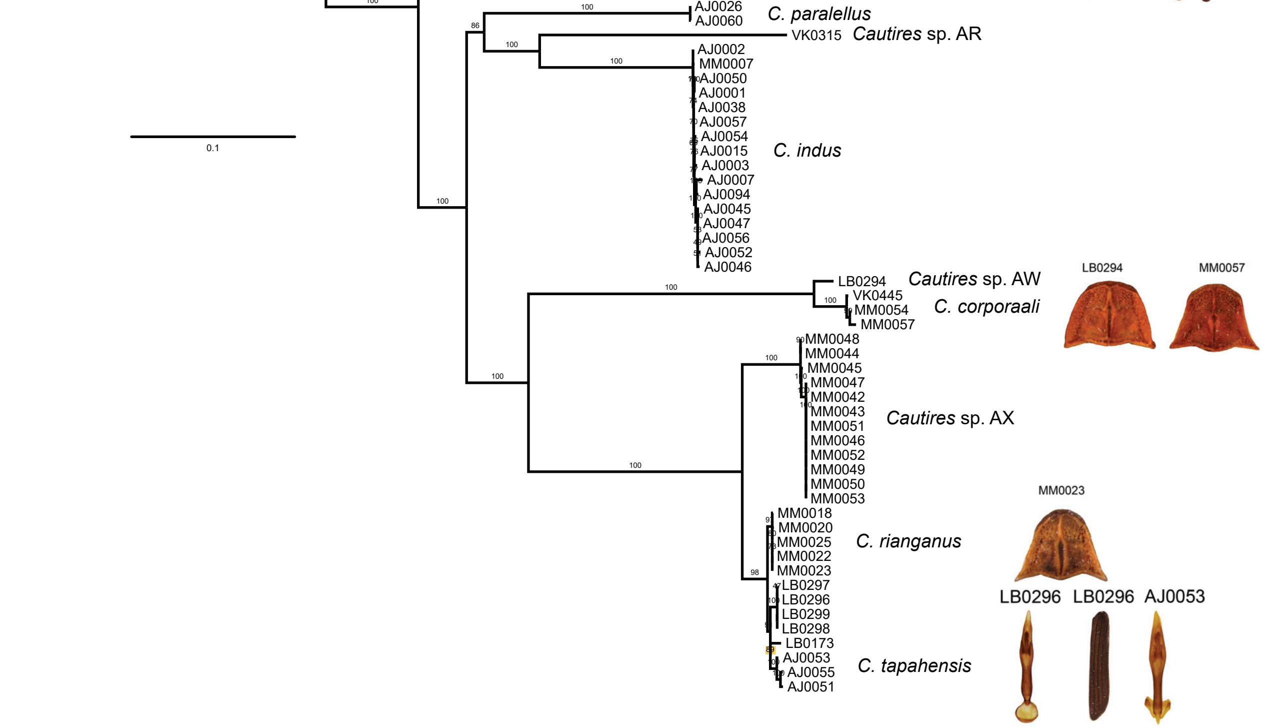

Supplementary Figure S1. Maximum likelihood analysis of full *Cautires* dataset including outgroup using IQ-TREE. Numbers on branches represent ultrafast boostrap values.

All photographs taken by © Authors

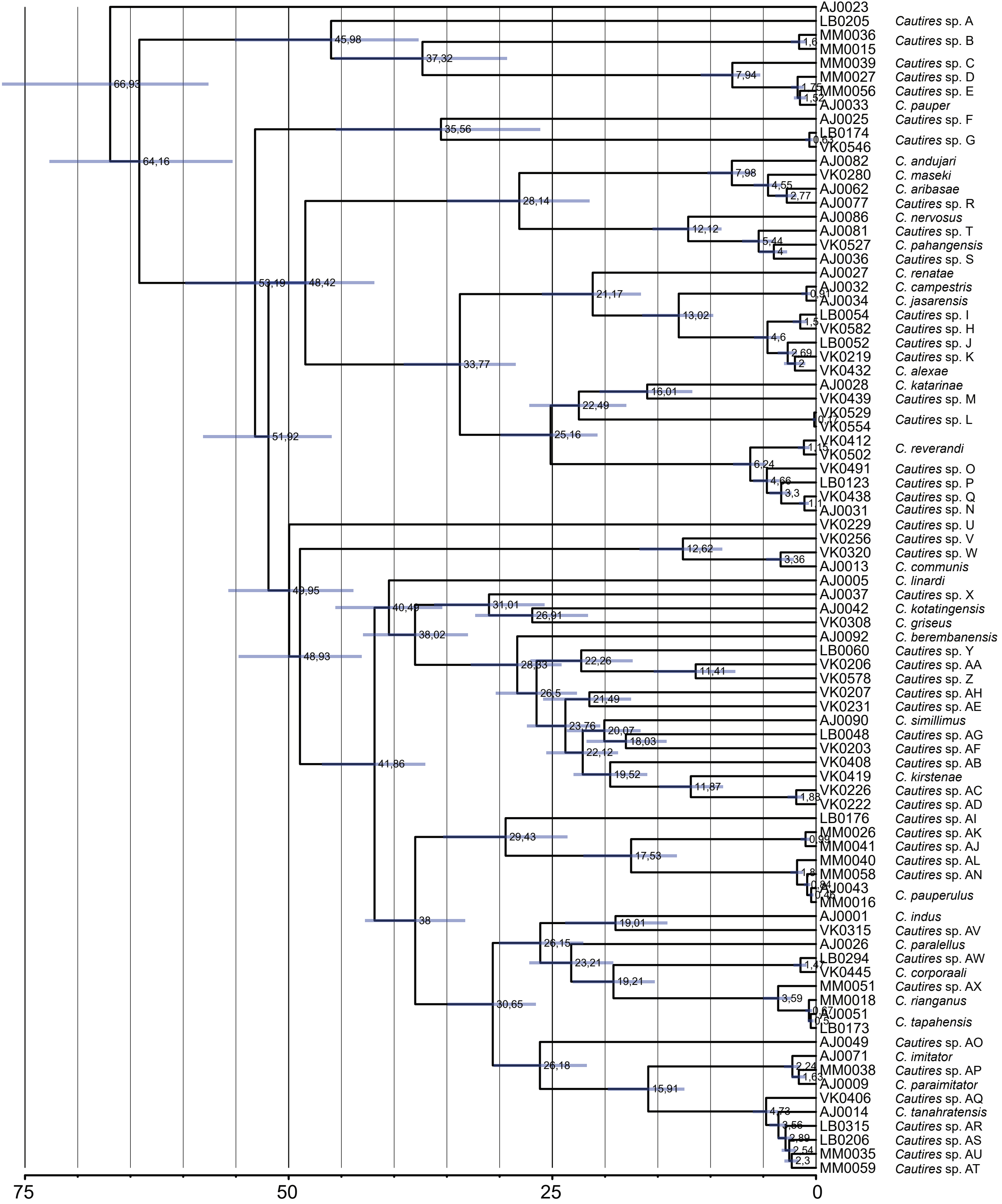

Supplementary Figure S2. Dated phylogenetic tree of *Cautires* using BEAST with constrained topology inferred from the maximum likelihood analysis of three-gene dataset.

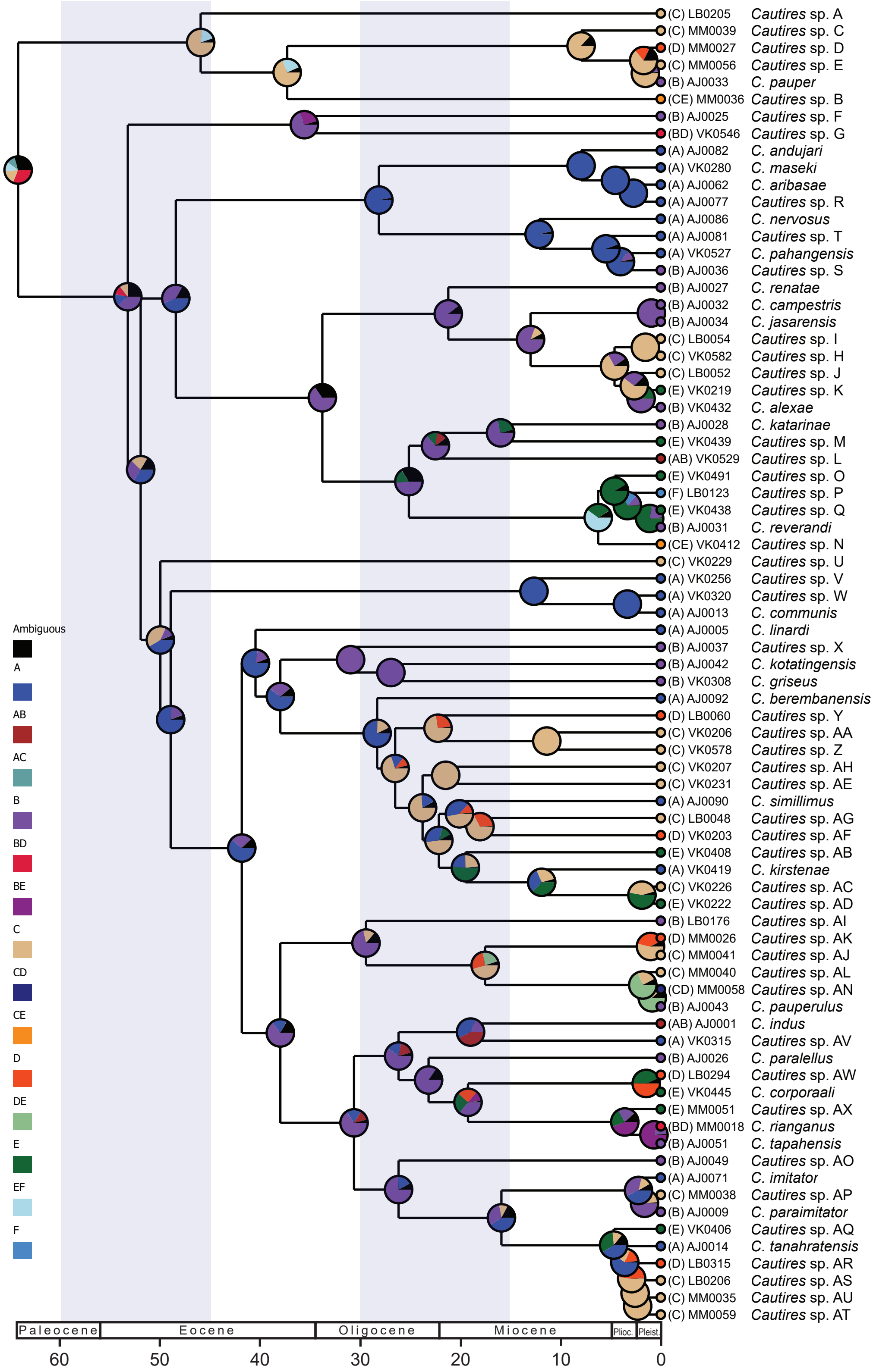

Supplementary Figure S3. Time calibrated maximum clade credibility tree of *Cautires* with reconstruction of specific geographic localities. Letters reffer to sampled areas: (A) Malaysia: Cameron Highland, (B) Malaysia: lowlands, (C) Sumatra: Tuju, (D) Sumatra: Merapi, (E) Sumatra: Sibayak, (F) Java and their respective combinations.

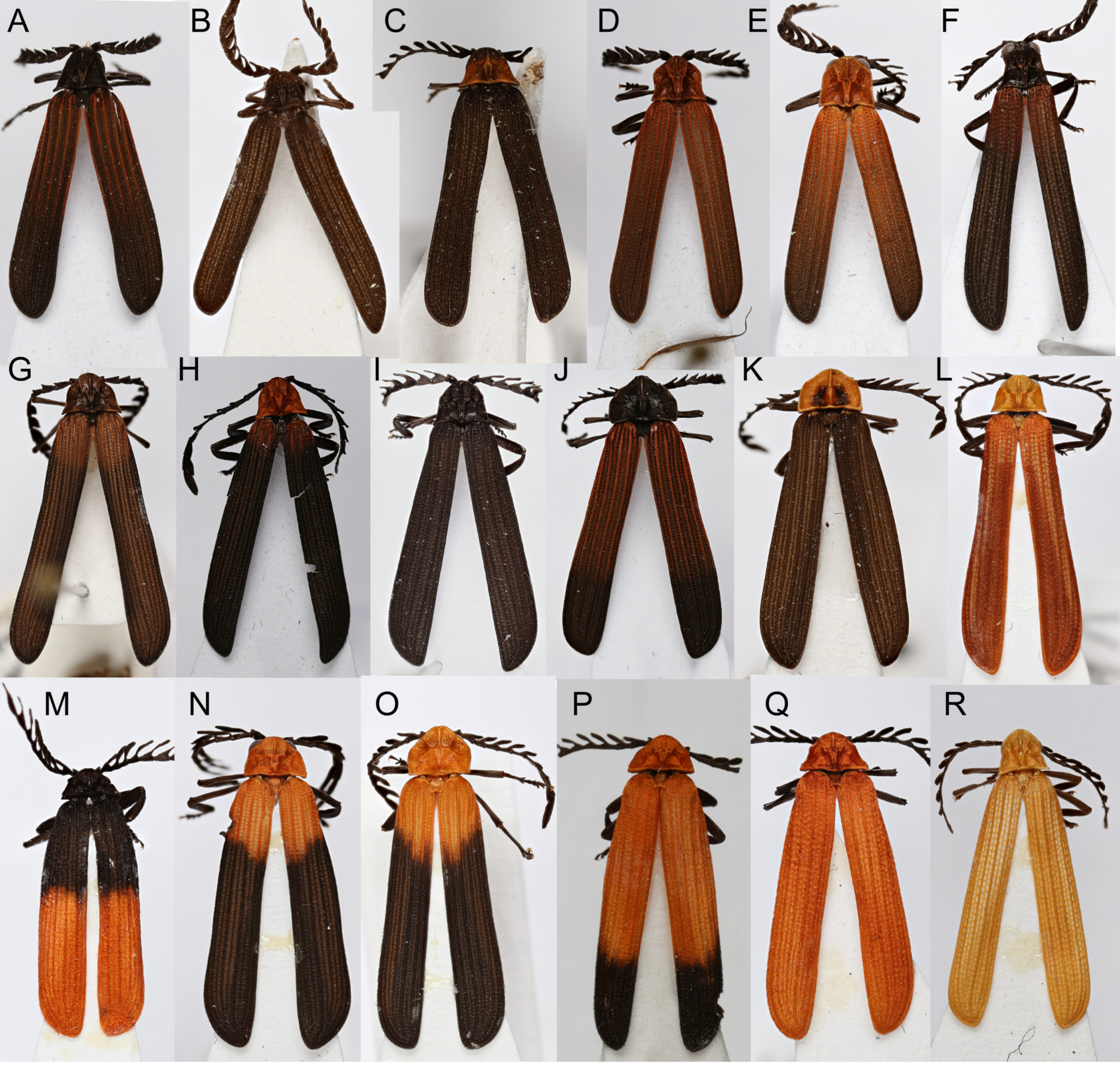

Supplementary Figure S4. Aposematic patterns of *Cautires* net-winged beetles in the Malay Peninsula and Sumatra. A–*Cautires indus* (Kirsch) (Voucher number AJ0046, Malaya: Tapah); B–*C. rianganus* (Pic) (LB0297, Sumatra: Talamau); C–ditto (MM0020, Sumatra: Maninjau): Rd Gua Musang); D–*C. reverandi* Pic (AJ0029: Rd Gua Musang); E–*C. katarinae* Jiruskova et al. (AJ0028, Malaya: Rd K. Raja to Gua Musang, 980 m); F–*C. arribasae* Jiruskova et al. (AJ0066, Malaya: Gunung Beremban); G–*Cautires* sp. T (LB0070, Malaya: Tanah Rata); H–ditto (AJ0074, Malaya: Tanah Rata); I–*C. communis* Jiruskova et al. (AJ0091, Malaya: Gunung Beremban); J–*C. pauperulus* Bourgeois (MM0043, Malaya: Tapah); K–*Cautires* sp. AK (MM0014, Sumatra: Maninjau); L–*Cautires* sp. AA (VK0228, Sumatra: Gunung Tujuh); M–*Cautires* sp. AE (VK0230, Sumatra: Gunung Tujuh); N–*Cautires* sp. K (VK0219, Sumatra: Gunung Sinabung); O–*Cautires* sp. AD (VK0222, Sumatra: Gunung Sinabung); P–*Cautires* sp. AH (MM060, Sumatra: Gunung Kerinci); Q–ditto (VK0210, Sumatra: Gunung Kerinci); R–*Cautires* sp. U (VK0214, Sumatra: Gunung Kerinci).

All photographs taken by © Authors
